# Unraveling the telomere-mitochondrial axis in colorectal cancer: Results from a prospectively followed cohort

**DOI:** 10.1186/s10020-026-01423-6

**Published:** 2026-02-21

**Authors:** Adrián Gil-Korilis, Jorge Ergui-Arbizu, Petr Hanák, Natálie Danešová, Kristýna Tomášová, Anna Valíčková, Josef Horák, Manuel Gentiluomo, Miroslav Levý, Soňa Křivonosková, Jan Král, Jiří Jungwirth, Ludmila Vodičková, Veronika Vymetálková, Amaya Azqueta, Daniele Campa, Pavel Vodička, Soňa Vodenková

**Affiliations:** 1 Department of Molecular Biology of Cancer, Institute of Experimental Medicine of the Czech Academy of Sciences, Prague, Czech Republic; 2 School of Medicine, University of Navarra, Pamplona, Spain; 3 School of Sciences, University of Navarra, Pamplona, Spain; 4 Biomedical Centre, Faculty of Medicine in Pilsen, Charles University, Pilsen, Czech Republic; 5 Institute of Biology and Medical Genetics, First Faculty of Medicine, Charles University, Prague, Czech Republic; 6 Department of Biology, Unit of Genetics, University of Pisa, Pisa, Italy; 7 Department of Surgery, First Faculty of Medicine, Charles University and Thomayer University Hospital, Prague, Czech Republic; 8 Department of Oncology, First Faculty of Medicine, Charles University and Thomayer University Hospital, Prague, Czech Republic; 9 Department of Internal Medicine, Second Faculty of Medicine, Charles University and Motol University Hospital, Prague, Czech Republic; 10 Department of Hepatogastroenterology, Institute for Clinical and Experimental Medicine, Prague, Czech Republic; 11 Institute of Physiology, First Faculty of Medicine, Charles University, Prague, Czech Republic; 12 Department of Gastroenterology, Libera Scientia, Prague, Czech Republic; 13 Biomedical Centre Martin, Jessenius Faculty of Medicine in Martin, Comenius University in Bratislava, Martin, Slovakia; 14 Department of Pharmaceutical Sciences, School of Pharmacy and Nutrition, University of Navarra, Pamplona, Spain

**Keywords:** Telomere-mitochondrial axis, Colorectal cancer, Mitochondrial DNA copy number, Telomere length, TaqMan, SYBR Green

## Abstract

**Background:**

Telomere shortening and mitochondrial dysfunction are well-known independent contributors to many diseases, but emerging evidence suggests a reciprocal relationship between the two processes. The role of the so-called telomere-mitochondrial axis in colorectal cancer (CRC) remains largely unknown.

**Methods:**

This prospective cohort study screened CRC patients who underwent surgery, from whom peripheral blood, intestinal mucosa, and tumor samples were collected. Colonoscopically confirmed cancer- and adenoma-free healthy individuals were screened as controls, from whom peripheral blood and intestinal mucosa samples were obtained. Relative mitochondrial DNA copy number (mtDNA-CN) and relative telomere length (RTL) were measured in all samples by real-time quantitative polymerase chain reaction and were further compared and correlated considering clinical data. Relative mtDNA-CN was quantified using both TaqMan probes and SYBR Green to compare both methods. Finally, multivariable analyses were conducted to investigate the association between both biomarkers and the risk of tumor recurrence and mortality.

**Results:**

A total of 166 CRC patients and 61 healthy individuals were included in the study. In TNM stage I patients, relative mtDNA-CN and RTL were negatively correlated with each other in intestinal mucosa (ρ = -0.77, *p* < 0.0001), tumor tissue (ρ = -0.41, *p* = 0.032), and the tumor-to-intestinal mucosa ratio (ρ = -0.39, *p* = 0.046). However, these associations disappeared with increasing TNM stage, suggesting a dysregulation of the telomere-mitochondrial axis in advanced disease. Higher relative mtDNA-CN in blood was associated with a lower risk of disease recurrence even after adjusting for multiple covariates (HR = 0.43, 95% CI 0.20–0.97, *p* = 0.041), highlighting its potential use as a prognostic tool. The quantification of mtDNA-CN performed by both methods -TaqMan probes and SYBR Green- was shown to be positively correlated (*p* < 0.01). Relative mtDNA-CN and RTL were found to be tissue-dependent in both CRC patients and healthy controls.

**Conclusions:**

This study provides a novel contribution to the understanding of the almost unexplored telomere-mitochondrial axis in CRC, highlighting its potential role in disease progression and prognosis.

**Graphical Abstract:**

Created with https://www.BioRender.com.

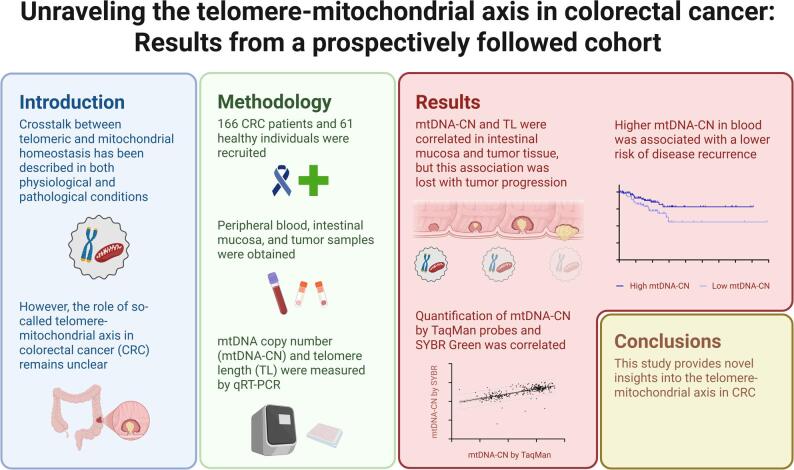

**Supplementary Information:**

The online version contains supplementary material available at 10.1186/s10020-026-01423-6.

## Introduction

Telomere attrition and mitochondrial dysfunction have largely been studied as separate contributors to aging and the development of age-related diseases, including most types of cancer. However, growing evidence suggests a link between these two processes (Zole and Ranka 2018). For this reason, recent research has focused on unraveling the molecular mechanisms underlying this crosstalk and on understanding their combined role in physiology and disease pathogenesis (Vaurs et al. 2024; Zhao et al. 2024; Nassour et al. 2023).

Mitochondria play a key role in maintaining cellular homeostasis, primarily by regulating energy metabolism, cell survival, and proliferation (Moro 2019). The mitochondrial proteome comprises over a thousand proteins, most of which are encoded by nuclear DNA (nDNA), while only thirteen are encoded by mitochondrial DNA (mtDNA) (Rath et al. 2021). The mitochondrial genome encodes the core subunits of the respiratory chain, which are crucial for oxidative phosphorylation and energy conversion (Rackham and Filipovska 2022).

Each human cell contains between several dozen and a few thousand mitochondria, with each mitochondrion housing two to ten copies of mtDNA (Alikhani et al. 2022). This results in a total of several hundred to over ten thousand mtDNA copies per cell (Wang et al. 2006). Consequently, the mtDNA copy number (mtDNA-CN), representing the total number of mtDNA molecules per cell, is widely used as a biomarker of mitochondrial function and amount (Castellani et al. 2020). It reflects physiological processes like aging, in which mtDNA-CN decreases with increasing age (Mengel-From et al. 2014). However, it is also associated with various pathological changes affecting different tissues, such as cardiovascular disease, chronic kidney disease, or neurodegenerative diseases (Castellani et al. 2020; Tong et al. 2024; Kazachkova et al. 2013). Additionally, alterations in mtDNA-CN have been observed across a range of human cancers (Reznik et al. 2016). For instance, increased intratumoral mtDNA-CN levels have been reported in head and neck (Kim et al. 2004), ovarian (Wang et al. 2006), and esophageal (Lin et al. 2010) cancers, whereas reduced mtDNA-CN has been found in hepatocellular (Yamada et al. 2006), renal (Xing et al. 2008), and breast (Yu et al. 2007) cancers. These findings suggest that alterations in mtDNA-CN may contribute to the initiation and progression of tumorigenesis, although the process is not fully understood (Abd Radzak et al. 2022).

During nDNA replication, a portion of the DNA at the 3' ends of linear chromosomes remains unreplicated (Olovnikov 1973). These ends, known as telomeres, consist of tandem repeats of the hexanucleotide sequence TTAGGG in mammals. Aging is associated with progressive telomere shortening (Chakravarti et al. 2021), which compromises chromosome integrity, as exposed chromosome ends are sensed as DNA double-strand breaks, triggering cellular senescence or programmed cell death (Tomasova et al. 2020). Similar to mtDNA-CN, telomere length (TL) varies in cancer: studies have mainly reported intratumoral telomere shortening in breast (Kammori et al. 2015), esophageal (Tahara et al. 2023), pancreatic, and liver (Sung et al. 2024) cancers, while telomere lengthening has been observed in neuroblastoma (Graham et al. 2024), melanoma, and lung (Rode et al. 2016) cancers. This highlights the dual role of TL in tumorigenesis, although the literature on this subject remains very heterogeneous, even within studies of the same tumor type (Campa et al. 2025).

Although TL shortening and mitochondrial dysfunction are well-established independent contributors to aging and disease, emerging evidence suggests a reciprocal relationship between these processes. For example, mitochondrial dysfunction has been shown to induce telomere fragility and shortening (Moro 2019), whereas the telomerase reverse transcriptase (TERT), the catalytic subunit of the telomerase enzyme complex (Dratwa et al. 2020), has been described as playing a role in safeguarding mtDNA from oxidative damage (Jahan et al. 2024; Marinaccio et al. 2023; Haendeler et al. 2009). Understanding these interactions has driven extensive research into the molecular mechanisms underlying them (Vaurs et al. 2024; Zhao et al. 2024; Nassour et al. 2023) and, ultimately, into the impact of the so-called telomere-mitochondrial axis on both health (Li et al. 2024) and disease (Kumar et al. 2020). The role of the telomere-mitochondrial axis has been investigated in various diseases, with growing attention in cancer research (Assalve et al. 2025). Although the disruption of telomere and mitochondrial homeostasis appears to be a hallmark of carcinogenesis (Gao et al. 2022), this axis remains largely unexplored in colorectal cancer (CRC), which ranks third in global cancer incidence and second in cancer-related mortality (Bray et al 2024). The understanding of the function of the telomere-mitochondrial axis in CRC is of particular significance for unraveling the molecular mechanisms underlying CRC and improving patient management.

In this study, we explored the telomere–mitochondrial axis from a clinical perspective, defining it as the relationship between relative mtDNA-CN and relative telomere length (RTL) across different tissues. Like that, we analyzed potential associations between both biomarkers in blood, intestinal mucosa, and tumor samples from 166 CRC patients and in blood and intestinal mucosa samples from 61 healthy control individuals. We also evaluated this relationship considering sociodemographic and clinicopathological variables. Finally, we analyzed the association of mtDNA-CN and RTL with the risk of CRC recurrence and mortality.

## Materials and methods

This study was conducted following the STROBE guidelines for reporting observational studies (Vandenbroucke et al. 2007). The complete item checklist is given in Supplementary Table 1, Additional File 1.

### Cohort of included individuals

This prospective cohort study screened CRC patients who underwent surgical treatment following a CRC diagnosis between January 2010 and December 2020 at the Department of Surgery, Thomayer University Hospital, Prague, Czech Republic. The primary inclusion criterion was the availability of the complete set of blood and tissue samples detailed below. The exclusion criteria encompassed CRC associated with well-defined hereditary syndromes (including Lynch and Cowden syndromes, familial adenomatous, and MUTYH-associated polyposis), a positive family history of CRC, the presence of concomitant or previous neoplastic diseases (including CRC), and an incomplete or unclear diagnosis. At baseline, the study patients provided self-reported information through a structured questionnaire on sociodemographic variables, including age, sex, height, weight, and diagnosis of diabetes. From this information, their body mass index (BMI) was calculated. Likewise, clinicopathological data related to their neoplastic process were collected by attending oncologists, including the dates of diagnosis and surgery, clinical stage (classified according to the Tumor-Node-Metastasis [TNM] system of the Union for International Cancer Control (UICC 2017) (Control and (UICC). TNM Classification of Malignant Tumours. 2017), presence of synchronous metastasis, tumor location (classified according to the International Statistical Classification of Diseases and Related Health Problems 10^th^ Revision [ICD-10] (World Health Organization 2016), stratifying in right colon [C18.0-18.5], left colon [C18.6–19], and rectum [C20]), histology, microsatellite instability (MSI) (classified according to the revised Bethesda Guidelines for hereditary nonpolyposis colorectal cancer [Lynch Syndrome] and Microsatellite Instability (Umar et al. 2004), stratifying in stable [0/5 microsatellite sequences of the panel mutated], low instability [1/5 microsatellite sequences mutated], and high instability [≥ 2/5 microsatellite sequences mutated]), and whether neoadjuvant chemotherapy was received. Several biological samples were collected at the time of preoperative examination (as described below), after which the patients were prospectively followed by attending oncologists to obtain information on the incidence of local recurrence, metachronous metastasis, and mortality until March 23^rd^, 2023.

Additionally, this study screened healthy individuals for control comparison. These individuals were recruited between June and December 2022 at the Department of Hepatogastroenterology, Institute for Clinical and Experimental Medicine, Prague, Czech Republic, and at the Department of Gastroenterology, Libera Scientia, Prague, Czech Republic. The inclusion criterion was the availability of intestinal tissue and blood samples, whereas the exclusion criteria included the presence of any abnormalities, such as polyps or neoplastic lesions, during routine colonoscopy screening. These people also provided self-reported information through a structured questionnaire on sociodemographic variables, including age, sex, height, and weight, from which their BMI was calculated.

All study participants signed a written consent to participate in the study and approved the use of their biological samples for genetic analyses. This study was conducted in compliance with the principles laid out in the World Medical Association Declaration of Helsinki and the Department of Health and Human Services Belmont Report. Participant information was coded to protect their identity. The inclusion of study participants was approved by the Ethics Committee of the Institute of Experimental Medicine, Czech Academy of Sciences, Prague, Czech Republic, and the Ethics Committee of the Institute for Clinical and Experimental Medicine and Thomayer University Hospital, Prague, Czech Republic (for CRC patients: Docket No. 06366/23 + 15,408/23, A-23–07; Docket No. 1004/16, G-16–06–38; Docket No. 786/09, G-09–04–09; protocol G-14–08–67) (for healthy individuals: Docket No. 6799/21, G-20–55; protocol G-17–03-02).

### Sample collection and processing, and DNA isolation

**Fig. 1:**
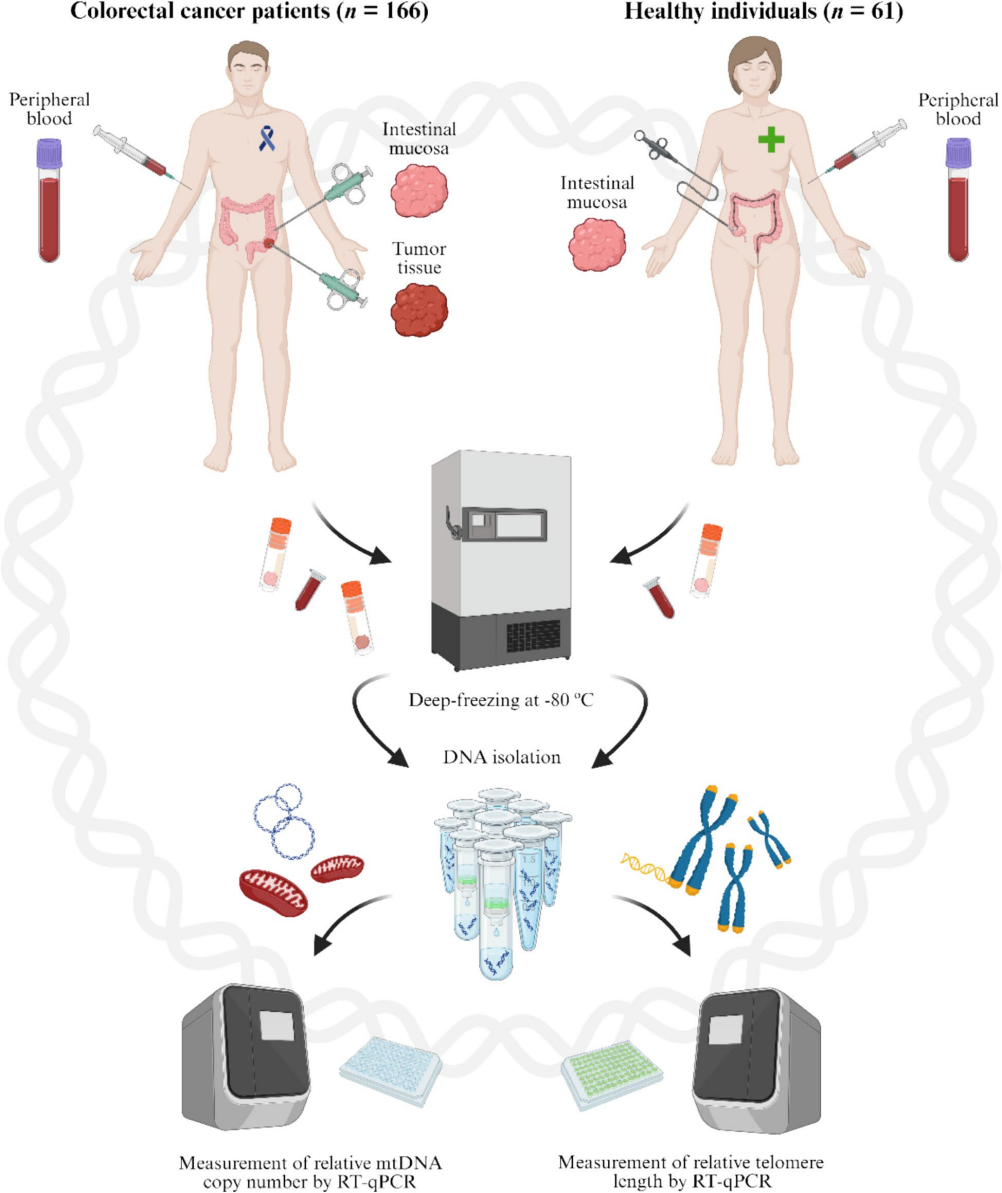
Schematic illustration of the sampling process and measured endpoints. RT-qPCR: real-time quantitative polymerase chain reaction. Created with BioRender.com

Peripheral blood collected by venipuncture into ethylenediaminetetraacetic acid (EDTA) tubes was obtained from all studied CRC patients at the time of preoperative examination, along with paired tumor and adjacent non-tumor intestinal mucosa biopsies (5–10 cm distant from the tumor) collected during surgery. Among rectal cancer patients, 42 (25.30% of the included cohort) had undergone neoadjuvant treatment at the time of sample collection. These patients were included after verifying that the therapy did not significantly affect the results (Additional Results, Additional file 1). From healthy individuals (controls), intestinal mucosa biopsies were taken during routine colonoscopies if no alterations (polyps or neoplastic lesions) were observed. After that, peripheral blood samples were collected as previously described. The blood samples were immediately transported at 4 °C to the Department of Molecular Biology of Cancer, Institute of Experimental Medicine, Czech Academy of Sciences, Prague, Czech Republic, for storage at −80 °C. Tissue samples were snap-frozen immediately after surgical/endoscopic resection and stored at −80 °C until their use for experimental analyses. The sampling process is illustrated schematically in Fig. 1.

Prior to DNA isolation, deep-frozen tissue samples were manually cut on a frozen plate placed on dry ice, and a small piece of each tissue sample was subsequently homogenized in RLT buffer (QIAGEN GmbH, Hilden, Germany) containing 1% β-mercaptoethanol (Sigma-Aldrich, Darmstadt, Germany) at 7669 g for 30 s using a MagNA Lyser Instrument (Roche Diagnostics GmbH, Mannheim, Germany). The DNA isolation from both blood and tissue samples was performed employing a DNeasy Blood & Tissue Kit (QIAGEN GmbH) according to the manufacturer’s instructions. The concentration of DNA was measured with a NanoDrop 2000 Spectrophotometer (Thermo Fisher Scientific, Waltham, USA), and samples were preserved at −20 °C.

### Measurement of relative mtDNA copy number

The relative mtDNA-CN was determined by real-time quantitative polymerase chain reaction (RT-qPCR) amplification in a QuantStudio™ 6 Flex Real-Time PCR System (Applied Biosystems^®^, Thermo Fisher Scientific, Waltham, USA). The results were analyzed with QuantStudio™ Real-Time PCR Software. To assess this determination, the mitochondria-specific gene NADH-ubiquinone oxidoreductase chain 1 (*MT-ND1*) was amplified and compared to the nuclear single-copy gene albumin (*ALB*) as the housekeeping gene. A duplex TaqMan probe-based approach (Applied Biosystems^®^, Thermo Fisher Scientific) was utilized, allowing for a parallel measurement of both target genes (Pfaffl 2001). A detailed description of the methodology can be found in the Additional Materials and Methods, Additional File 1.

Furthermore, relative mtDNA-CN was also quantified with an alternative method based on a monochromatic duplex RT-qPCR approach employing SYBR Green, as previously described (Gentiluomo et al. 2020), to compare it with the method using TaqMan probes (Additional Materials and Methods, Additional File 1). Primer sequences are listed in Supplementary Table 2, Additional File 1.

### Measurement of relative telomere length

The RTL of the samples was also determined by RT-qPCR amplification. Canonical 5’-TTAGGG-3’ telomere motif repeats were amplified and compared to *ALB* as the housekeeping gene. A monochromatic duplex RT-qPCR approach was employed, measuring both target and reference genes together in a single reaction in two sequential blocks of RT-qPCR, head to tail one to the other. SYBR Green-based SYTO™ 9 green fluorescent dye (Invitrogen™, Thermo Fisher Scientific) was used for the detection of both amplification products (Additional Materials and Methods, Additional File 1). Primer sequences are listed in Supplementary Table 2, Additional File 1. *ALB* primers were constructed with a GC-clamp at the 5′ end to increase the melting temperature (Cawthon 2009).

### Data (pre)processing

Both the relative mtDNA-CN and RTL were measured and compared across the three types of tissues from CRC patients: blood, non-tumor intestinal mucosa, and tumor. A ΔCt_*tumor*_/ΔCt_*mucosa*_ ratio was calculated for each patient to normalize the tumor results. Subsequently, biomarkers of individual tissues and the calculated ratios were compared according to the baseline sociodemographic and clinicopathological characteristics of the patients. For these analyses, only the relative mtDNA-CN measured with TaqMan probes was considered. The relative mtDNA-CN measured with SYBR Green was only employed to compare both quantification methods. Additionally, relative mtDNA-CN and RTL measurements from blood and intestinal mucosa samples obtained from healthy individuals (controls) were included in the analysis for comparison. Results were graphically plotted with violin plots, and -ΔCt values were shown instead of ΔCt to facilitate interpreting the results.

### Statistical analysis

To estimate the sample size required for the study, a power analysis was conducted based on the main objective—assessing the correlation between relative mtDNA-CN and RTL in the tissues of the participants. The power analysis was performed using G*Power Software version 3.1.9.7 (Heinrich-Heine-Universität Düsseldorf, Düsseldorf, Germany) for a correlation—difference from constant (one sample case) using the probability of an α error of 0.05, a power of 0.95, and a weak effect size (ρ = 0.3) for a two-tailed test. Based on these assumptions, the required sample size was calculated to be 138.

The Shapiro–Wilk test was used to explore the normality of quantitative variables. Variables following the normal distribution were described as arithmetic means ($\overline{x }$) and standard deviations (*s*), while those deviating from normality were described as medians (P_50_) and interquartile ranges (IQR). Qualitative variables were reported by frequencies and percentages.

Fisher’s exact test was utilized to compare categorical variables across groups. The unpaired Student *t-*test was employed to compare the means between two independent groups, whereas the Mann–Whitney *U* test was used for non-parametric data. The paired Student *t-*test was employed to compare the means between two matched groups. Ordinary one-way analysis of variance (ANOVA) was utilized to compare the means among three or more independent groups, whereas the Kruskal–Wallis test was applied for non-parametric data. The Friedman test was used to compare the mean ranks across three or more matched groups. When *p*-values were statistically significant, ANOVA, Kruskal–Wallis, and Friedman tests were followed by post hoc pairwise group comparisons, adjusting for multiple comparisons using the Benjamini and Hochberg method to control the false discovery rate (FDR) at 5%.

The correlation between variables was assessed by the calculation of Spearman’s rho (ρ) correlation coefficient and graphically plotted by linear regression, along with 95% confidence intervals (CI) and 95% prediction intervals (PI).

Survival analyses were performed considering relapse-free survival (RFS) and overall survival (OS) as the primary endpoints. RFS was defined as the elapsed time from the date of diagnosis to the date of the first evidence of local recurrence or metachronous metastasis, while OS was defined as the time from the date of diagnosis to the date of death from any cause. Censorship was established if CRC patients were free of local recurrence, metachronous metastasis, and/or were alive at the last follow-up date. All cases were regularly monitored until March 23^rd^, 2023. When necessary, patients were categorized into two groups according to the variable of interest, with one group having values above the median and the other group below the median. Survival was graphically plotted using Kaplan–Meier curves, and survival curves were compared employing the log-rank test. The median RFS and OS times, as well as the probabilities of RFS and OS at specific time points, were determined.

To further understand the relationship between mtDNA-CN/RTL and recurrence/mortality, multivariable Cox regression analyses were performed. Firstly, unadjusted models were constructed to explore the impact of several covariates on recurrence/mortality and to identify independent predictors as possible confounders. Secondly, the association between mtDNA-CN/RTL and recurrence/mortality was estimated through different lines of modeling adjusting for age (as continuous variable), sex (female [reference category], male), BMI (< 25 kg/m^2^ [reference category], ≥ 25 kg/m^2^), diabetes (no [reference category], yes), TNM stage (I [reference category], II, III, or IV), tumor location (right colon [reference category], left colon, rectum), tumor histology (mucinous [reference category], mixed, other), MSI (stable [reference category], low instability, high instability, undetected), or neoadjuvant chemotherapy received (no [reference category], yes). The specific models used are listed in the respective results table. As for the survival analysis, person-time was counted from the date of diagnosis for every CRC patient until the date of the first evidence of local recurrence or metachronous metastasis, date of death from any cause (both different endpoints for the same patient), last follow-up date or March 23^rd^, 2023 (censoring), whichever came first. The effect modification of an association between mtDNA-CN/RTL and both endpoints was evaluated by introducing interaction terms between covariates and biomarkers into the Cox models, with significance assessed by the Wald test. Finally, the results were presented as hazard ratios (HR) and 95% CI, with the below-median group as reference.

All *p-*values were two-tailed, with *p* < 0.05 considered statistically significant. Significance levels were denoted as follows: *, *p* < 0.05; **, *p* < 0.01; ***, *p* < 0.001; ****, *p* < 0.0001. The 95% CI was calculated as 1.96 × standard error of the mean (SEM).

All analyses were performed using GraphPad Prism software version 9.5.1 (GraphPad Software Inc., San Diego, CA, USA), STATA software version 15.1 (Stata Corp, College Station, TX, USA), and R software version 4.3.1 (The R Foundation) along with various R packages including dplyr, ggfortify, ggplot2, ggprism, ggpubr, ggsignif, outliers, readxl, rstatix, stringr, and tidyr.

## Results

### Baseline characteristics of study participants

**Fig. 2:**
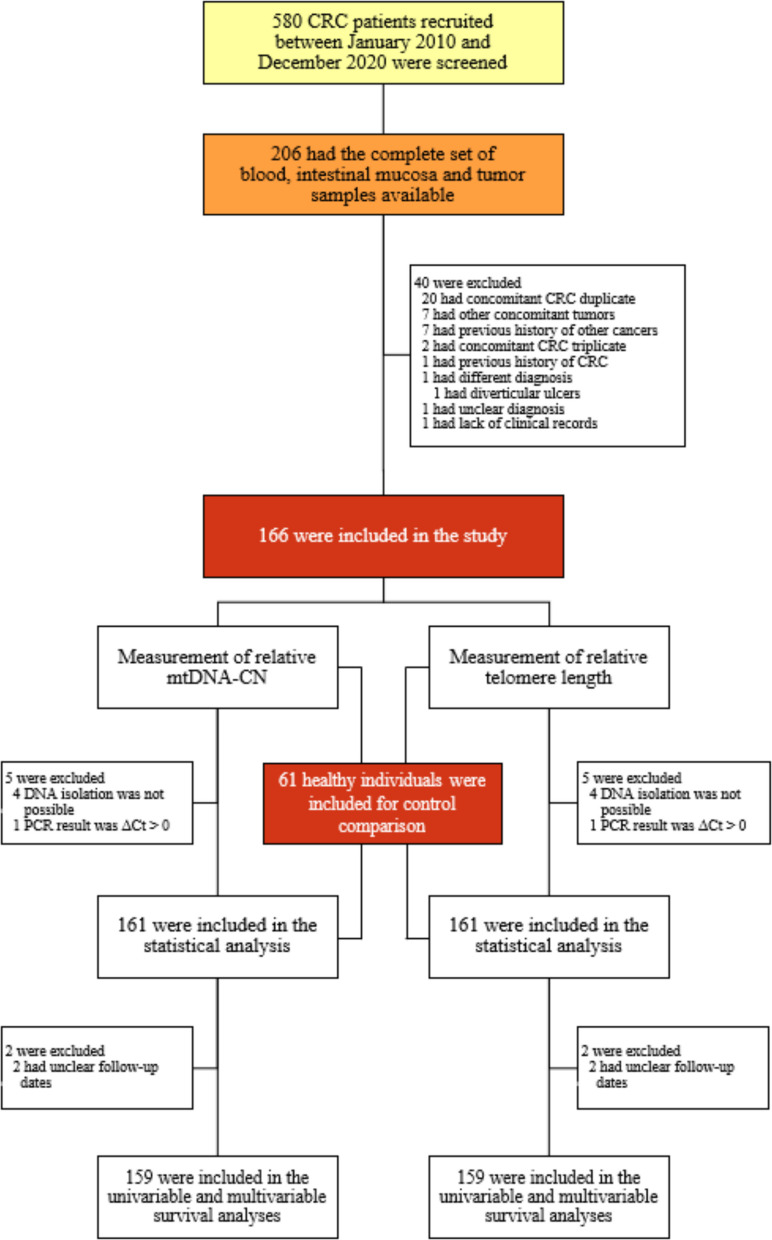
Flowchart of the study. The flowchart depicts the patients included in this study and the endpoints measured in their biological samples. CRC: colorectal cancer; Ct: cycle threshold; mtDNA-CN: mitochondrial DNA copy number; PCR: polymerase chain reaction

A total of 580 CRC patients were screened. Out of 206 who met the inclusion criterion, 166 (80.58%) were finally included in this study (Fig. 2). The sociodemographic and clinicopathological characteristics at baseline of the included patients are described in Table 1, along with the characteristics of the excluded patients (*n* = 40). A comparison between the included and excluded cohorts revealed that only the proportion of patients living with diabetes was statistically different. Therefore, the exclusion of these patients was non-informative in terms of overall cohort characteristics. Additionally, 61 healthy individuals were included for control comparison. These controls were significantly younger than the included patient cohort, and a higher proportion of female individuals was observed in the control group (Table 2).

**Table 1:** Sociodemographic and clinicopathological characteristics at baseline of the included and excluded cohorts of CRC patients

	**Included cohort** *n* = 166	**Excluded cohort**^**a**^ *n* = 40	***p*-value**
Age (years) [P_50_ (IQR)]	69 (60–75)	72 (65–76)	0.06
Sex [*n* (%)]			1.00
Female	56 (33.73)	13 (32.50)	
Male	110 (66.27)	27 (67.50)	
BMI (kg/m^2^) ($\overline{x }\pm s$)	27.93$\pm$4.74	26.90$\pm$4.88	0.24
Diabetes [*n* (%)]	38 (22.89)	16 (40.00)	**0.04**
TNM stage^b,c^ [*n* (%)]			0.35
0	0 (0.00)	1 (2.50)	
I	29 (17.47)	5 (12.50)	
II	61 (36.75)	10 (25.00)	
III	47 (28.31)	10 (25.00)	
IV	27 (16.27)	7 (17.50)	
Synchronous metastasis [*n* (%)]	27 (16.27)	7 (17.50)	0.61
Tumor location^b,d,e^ [*n* (%)]			0.09
Right colon	40 (24.10)	16 (36.33)	
Left colon	73 (43.98)	12 (27.27)	
Rectum	53 (31.93)	16 (36.33)	
Histology^b^ [*n* (%)]			0.65
Mucinous	18 (10.48)	6 (15.00)	
Mixed	13 (7.83)	2 (5.00)	
Other	133 (80.12)	29 (72.50)	
MSI^b,f^ [*n* (%)]			0.13
Stable	94 (56.63)	15 (37.50)	
Low instability	5 (3.01)	0 (0.00)	
High instability	6 (3.61)	4 (10.00)	
Neoadjuvant chemotherapy received^b^[*n*(%)]	43 (25.90)	10 (26.32)	1.00

Data information: The medians of age of the two groups were compared using the Mann–Whitney *U* test. The means of BMI of the two groups were compared using the unpaired Student *t-*test. Differences between the two groups in the remaining categorical variables were assessed using Fisher’s exact test

^a^CRC patients who met the exclusion criteria

^b^Values may not add up to 100% of available patients due to missing data

^c^Staging was based on pathological staging according to the TNM system of the UICC (Control and (UICC 2017). TNM Classification of Malignant Tumours. 2017)

^d^Stratification was performed according to the ICD-10 (World Health Organization 2016), categorizing in right colon (C18.0-18.5), left colon (C18.6–19), and rectum (C20)

^e^The excluded cohort is calculated over 44 due to multiple disease in some patients

^f^Stratification was performed according to the revised Bethesda Guidelines for hereditary nonpolyposis colorectal cancer (Lynch Syndrome) and Microsatellite Instability (Umar et al. 2004), categorizing in stable (0/5 microsatellite sequences of the panel mutated), low instability (1/5 microsatellite sequences mutated), and high instability (≥ 2/5 microsatellite sequences mutated).

*BMI* body mass index, *CRC* colorectal cancer, *ICD-10* International Statistical Classification of Diseases and Related Health Problems 10^th^ Revision, *IQR* interquartile range, *MSI* microsatellite instability, P_50_: median, *s *standard deviation, *TNM* Tumor-Node-Metastasis, *UICC* Union for International Cancer Control, $\overline{x }$: arithmetic mean

**Table 2:** Sociodemographic characteristics at baseline of the included cohort of CRC patients and controls

	**CRC patients** *n* = 166	**Controls** *n* = 61	***p*-value**
Age (years) [P_50_ (IQR)]	69 (60–75)	50 (45–57)	**< 0.0001**
Sex [*n* (%)]			**0.002**
Female	56 (33.73)	35 (57.38)	
Male	110 (66.27)	26 (42.62)	
BMI (kg/m^2^) ($\overline{x }\pm s$)	27.93$\pm$4.73	27.55$\pm$4.66	0.60

Data information: The medians of age of the two groups were compared using the Mann–Whitney *U* test. The means of BMI of the two groups were compared using the unpaired Student *t-*test. The difference between the sexes of the two groups was assessed using Fisher’s exact test

*BMI* body mass index, *CRC* colorectal cancer, *IQR* interquartile range, *P*_*50*_: median, *s* standard deviation,$\overline{x }$: arithmetic mean

### Stage-dependent correlation between relative mtDNA copy number and relative telomere length across tissue types

There was a weak, significant negative correlation between relative mtDNA-CN and RTL in intestinal mucosa and tumors from CRC patients (Table 3). These correlations were stronger in TNM I patients and weakened until they became non-significant with increasing tumor stage. A significant negative correlation between both biomarkers was also found in the intestinal mucosa from controls. In contrast, no correlations were found in blood from CRC patients or controls.

**Table 3:** Correlation between relative mtDNA-CN and RTL across tissue types according to TNM stages in CRC patients and controls

	***N***	**ρ correlation coefficient**	***p*-value**
Blood from CRC patients			
Whole cohort	160	−0.11	0.18
TNM I	27	−0.04	0.85
TNM II	59	−0.11	0.39
TNM III	46	−0.20	0.18
TNM IV	26	−0.10	0.64
Intestinal mucosa from CRC patients			
Whole cohort	160	−0.28	**0.0003**
TNM I	27	−0.77	**< 0.0001**
TNM II	59	−0.39	**0.0023**
TNM III	46	0.04	0.77
TNM IV	26	−0.13	0.52
Tumor from CRC patients			
Whole cohort	160	−0.18	**0.02**
TNM I	27	−0.41	**0.032**
TNM II	59	−0.02	0.89
TNM III	46	−0.06	0.71
TNM IV	26	−0.36	0.07
Ratio tumor/intestinal mucosa from CRC patients			
Whole cohort	160	−0.01	0.87
TNM I	27	−0.39	**0.046**
TNM II	59	0.12	0.37
TNM III	46	0.06	0.70
TNM IV	26	−0.06	0.78
Controls			
Blood	61	−0.24	0.07
Intestinal mucosa	61	−0.26	**0.045**

The TNM staging was classified according to the UICC (Control and UICC 2017).

TNM Classification of Malignant Tumours. 2017). *CRC* colorectal cancer, *mtDNA-CN* mitochondrial DNA copy number, *RTL* relative telomere length, *TNM* Tumor-Node-Metastasis system, *UICC* Union for International Cancer Control

### Blood mtDNA copy number as a potential biomarker for relapse-free survival

A total of 159 CRC patients were included in the survival analysis when employing relative mtDNA-CN and RTL data (for further omission of patients see Fig. 2). Given the small number of excluded cases, no imputation or other missing data techniques were applied. The mean follow-up time was 27.55 months (*s* = 20.94). During the follow-up, 31 patients relapsed (67.74% men) with a mean RFS of 14.75 months (*s* = 9.47), and 26 patients died (73.08% men) with a mean OS of 18.14 months (*s* = 12.04). For the survival analysis, patients were stratified into two groups according to the median of the variable of interest.

**Fig. 3:**
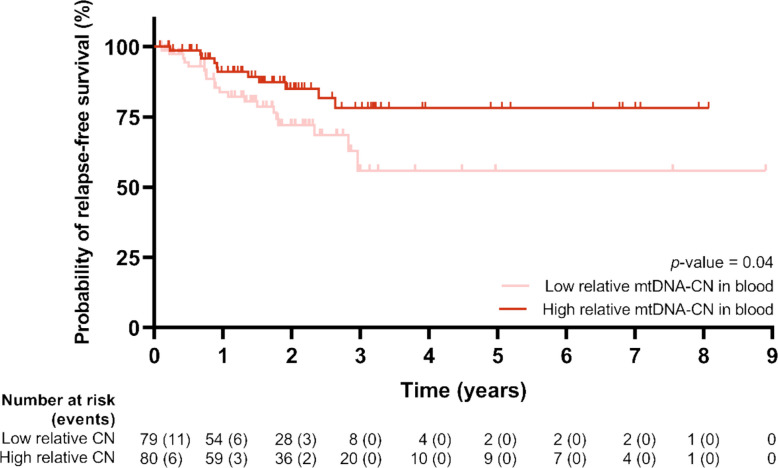
Kaplan–Meier curves for RFS when stratifying CRC patients by relative mtDNA-CN in blood Data information: Patients were stratified into two groups according to the median of the variable. The number-at-risk table is shown below the curves. The *p*-value by the log-rank test is displayed. CN: copy number; CRC: colorectal cancer; mtDNA-CN: mitochondrial DNA copy number

The results for relative mtDNA-CN in blood regarding RFS were statistically significant and clinically relevant, highlighting the potential of blood as a minimally invasive source of biomarkers. Specifically, when stratifying CRC patients into two groups according to their relative mtDNA-CN in blood (< P_50_, *n* = 79; > P_50_, *n* = 80), relapse occurred in 20 patients (25.32%) in the < P_50_ group and in 11 (13.75%) in the > P_50_ group, and the log-rank test revealed that the survival curves of both groups were different from each other (*p* = 0.04) (Fig. 3). Among the total of 31 patients who experienced recurrence, 17 patients (54.84%) recurred within the first year after diagnosis, 9 patients recurred in the second year (29.03%), and 3 patients recurred in the third year (9.68%). Median survival times for RFS were not reached in either group (Supplementary Table 3, Additional File 1).

The remaining survival analyses of RFS and OS considering relative mtDNA-CN and RTL across different tissue types did not yield any significant results (Additional Results, Supplementary Figs. 1 and 2, and Supplementary Tables 3 and 4, all found in Additional File 1).

### Multivariable survival analysis supports the prognostic value of blood mtDNA copy number

The multivariable survival analyses between mtDNA-CN and recurrence/mortality were based on 326.87/359.52 person-years of follow-up, whereas the analyses between RTL and recurrence/mortality were based on 327.22/359.66 person-years. Unadjusted models showed that TNM stage and MSI could function as independent predictors for recurrence and mortality in this sample (Table 4). The multivariable analysis revealed that having a higher relative mtDNA-CN in blood at diagnosis was significantly associated with a lower risk of recurrence (HR = 0.43, 95% CI 0.20–0.97, *p* = 0.041 in the fully adjusted model) (Table 5). The association between the risk of recurrence and relative mtDNA-CN in blood as a continuous variable was HR = 0.77 (95% CI 0.42–1.43, *p* = 0.42) in the fully adjusted model (Supplementary Table 5, Additional File 1). The interactions between covariates and relative mtDNA-CN in blood are shown in Supplementary Table 6, Additional File 1.

**Table 4:** Identification of independent predictors as potential confounders for multivariable models

Variable	Unadjusted HR (95% CI) for risk of recurrence	Unadjusted HR (95% CI) for risk of mortality
Age	0.97 (0.95–1.00)	0.99 (0.96–1.03)
Sex		
Female (ref.)	1.00	1.00
Male	1.11 (0.52–2.37)	1.45 (0.61–3.44)
BMI		
< 25 kg/m^2^ (ref.)	1.00	1.00
≥ 25 kg/m^2^	1.53 (0.62–3.74)	1.25 (0.50–3.12)
Diabetes		
No (ref.)	1.00	1.00
Yes	0.67 (0.26–1.74)	1.14 (0.46–2.86)
TNM stage^a^		
I (ref.)	1.00	1.00
II	2.16 (0.46–10.18)	3.62 (0.44–30.15)
III	3.33 (0.73–15.23)	5.31 (0.65–43.27)
IV	8.69 (1.88–40.25)**	14.47 (1.84–113.84)*
Synchronous metastasis		
No (ref.)	1.00	1.00
Yes	3.56 (1.65–7.68)**	3.54 (1.60–7.85)**
Tumor location^b^		
Right colon (ref.)	1.00	1.00
Left colon	0.58 (0.21–1.55)	0.49 (0.19–1.27)
Rectum	1.40 (0.57–3.45)	0.58 (0.22–1.52)
Tumor histology		
Mucinous (ref.)	1.00	1.00
Mixed	-	2.96 (0.31–28.72)
Other	1.06 (0.32–3.50)	2.02 (0.27–15.10)
MSI^c^		
Stable (ref.)	1.00	1.00
Low instability	1.57 (0.21–11.91)	4.96 (1.09–22.67)*
High instability	4.09 (1.20–14.02)*	3.32 (0.74–14.83)
Undetected	0.82 (0.37–1.81)	0.76 (0.31–1.86)
Neoadjuvant chemotherapy received		
No (ref.)	1.00	1.00
Yes	1.63 (0.78–3.40)	0.83 (0.33–2.07)

^a^Staging was based on pathological staging according to the TNM system of the UICC (Control and UICC 2017). TNM Classification of Malignant Tumours. 2017)

^b^Stratification was performed according to the ICD-10 (World Health Organization 2016), categorizing in right colon (C18.0-18.5), left colon (C18.6–19), and rectum (C20)

^c^Stratification was performed according to the revised Bethesda Guidelines for hereditary nonpolyposis colorectal cancer (Lynch Syndrome) and Microsatellite Instability (Umar et al. 2004), categorizing in stable (0/5 microsatellite sequences of the panel mutated), low instability (1/5 microsatellite sequences mutated), and high instability (≥ 2/5 microsatellite sequences mutated)

*, *p* < 0.05; **, *p* < 0.01

*BMI* body mass index, *CI* confidence interval, *HR* hazard ratio, *ICD-10* International Statistical Classification of Diseases and Related Health Problems 10^th^ Revision, *MSI* microsatellite instability, *TNM* Tumor-Node-Metastasis, *UICC* Union for International Cancer Control

**Table 5:** Association between relative mtDNA-CN in blood and risk of recurrence (local recurrence or metachronous metastasis) in CRC patients

	***N***	**Person-years**	**Number of recurrent cases**	**Incidence rate (per 10 person-years)**	**Crude model**	**Model 1**^**a**^	**Model 2**^**b**^
					**HR (95% CI)**		
Low mtDNA-CN (ref.)	79	142.04	20	1.41	1.00	1.00	1.00
High mtDNA-CN	80	184.83	11	0.60	0.47 (0.22–0.97)*	0.43 (0.20–0.91)*	0.43 (0.20–0.97)*

^a^ Adjusted for age and sex

^b^ Fully adjusted for age, sex, BMI, diabetes, TNM stage, tumor location, tumor histology, MSI, and neoadjuvant chemotherapy received

*, *p* < 0.05. Patients were stratified into two groups according to the median of the variable. The TNM staging was classified according to the UICC (Control and (UICC 2017). TNM Classification of Malignant Tumours. 2017). Tumor location was classified according to the ICD-10 (World Health Organization 2016), stratifying in right colon (C18.0-18.5), left colon (C18.6–19), and rectum (C20). MSI was classified according to the revised Bethesda Guidelines for hereditary nonpolyposis colorectal cancer (Lynch Syndrome) and Microsatellite Instability (Umar et al. 2004), stratifying in stable (0/5 microsatellite sequences of the panel mutated), low instability (1/5 microsatellite sequences mutated), and high instability (≥ 2/5 microsatellite sequences mutated)

*BMI* body mass index, *CI* confidence interval, *CRC* colorectal cancer, *HR* hazard ratio, *ICD-10* International Statistical Classification of Diseases and Related Health Problems 10^th^ Revision, *MSI* microsatellite instability, *mtDNA-CN* mitochondrial DNA copy number, *TNM* Tumor-Node-Metastasis system, *UICC* Union for International Cancer Control

The analysis considering the relative mtDNA-CN in other tissues, as well as the multivariable analyses for relative mtDNA-CN and mortality, RTL and recurrence, and RTL and mortality did not yield any significant results (Supplementary Tables 7–13, Additional File 1).

### Relative mtDNA copy number: Method comparison, tissue-specific patterns, and correlations

**Fig. 4:**
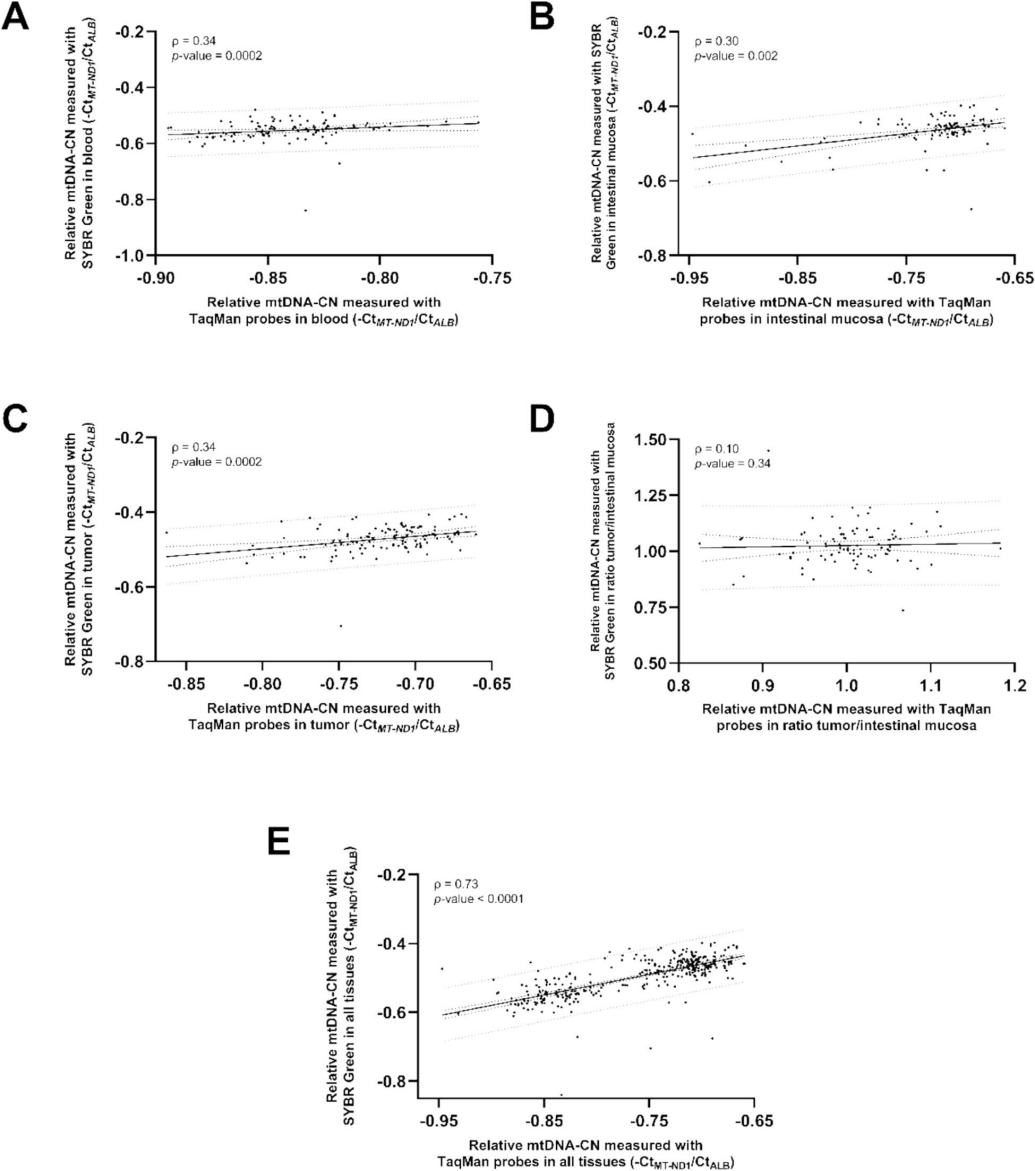
Correlation between relative mtDNA-CN measured with SYBR Green and TaqMan probes in blood (**A**), non-tumor intestinal mucosa (**B**), tumor (**C**), ratio tumor/intestinal mucosa (**D**), and all tissues (blood, non-tumor intestinal mucosa, and tumor) (**E**) from CRC patients Data information: Sample sizes of the groups: *n*_blood_ = 113, *n*_intestinal mucosa_ = 106, *n*_tumor_ = 117, *n*_tumor/intestinal mucosa_ = 91, *n*_all tissues_ = 336. In each graph, both Spearman’s rho (ρ) correlation coefficient and the *p*-value are shown, together with the 95% confidence intervals (dark dotted lines) and the 95% prediction intervals (gray dotted lines). CRC: colorectal cancer; Ct: cycle threshold; mtDNA-CN: mitochondrial DNA copy number

**Fig. 5:**
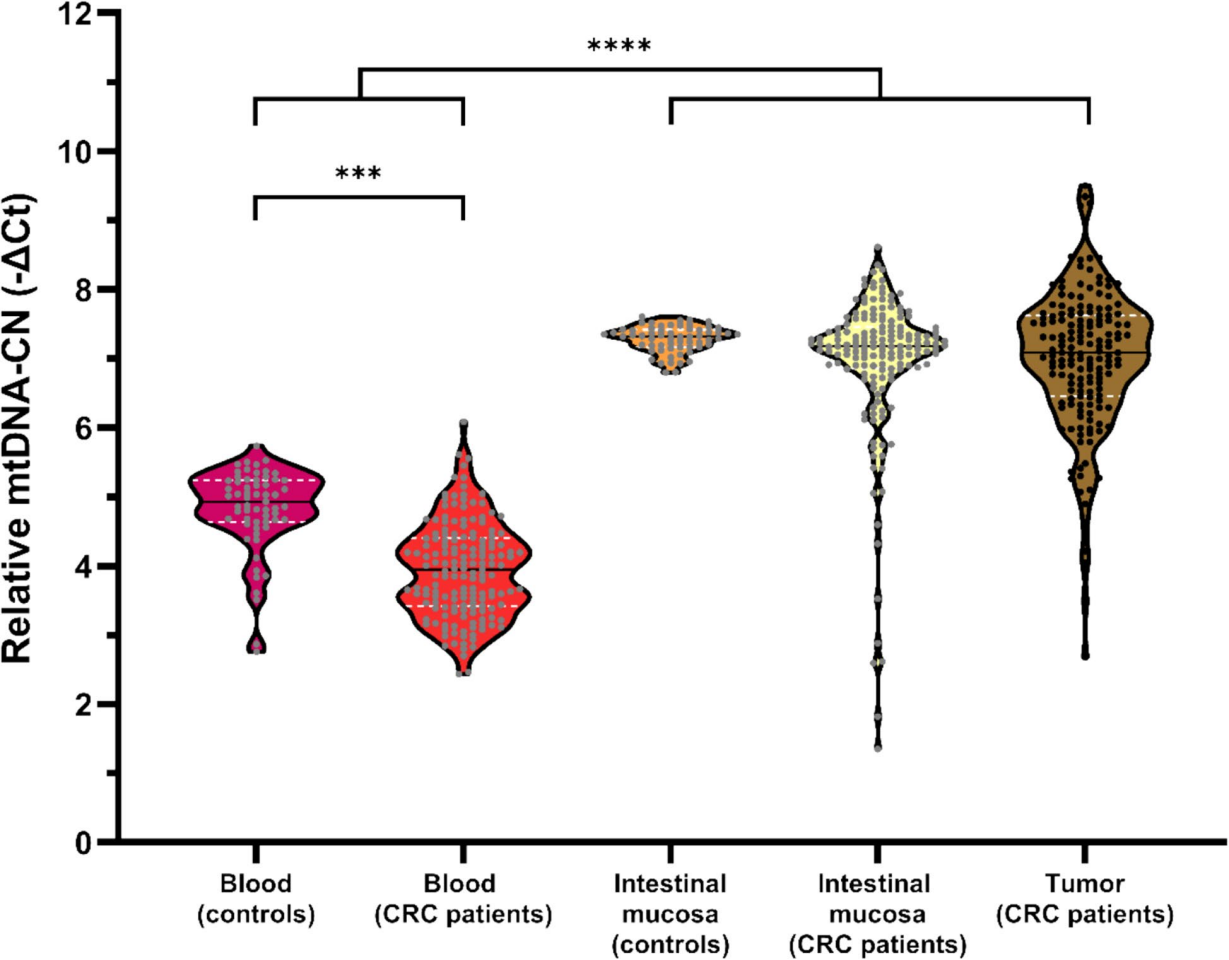
Relative mtDNA-CN according to tissue type Data information: Sample sizes of the groups: *n*_blood_ = *n*_intestinal mucosa_ = *n*_tumor_ = 161 for CRC patients, *n*_blood_ = *n*_intestinal mucosa_ = 61 for controls. Statistical differences were assessed with the Friedman test followed by pairwise multiple comparisons correcting the resultant *p*-values with the method of Benjamini and Hochberg controlling the FDR at 5% to compare the blood, intestinal mucosa, and tumor groups from CRC patients; with the Kruskal–Wallis test followed by the same multiple comparisons to compare the mean ranks of the blood, intestinal mucosa, and tumor groups from CRC patients to the mean rank of the blood and intestinal mucosa groups from controls as control groups; and with the paired Student *t-*test to compare the blood and intestinal mucosa groups from controls. ***, *p* < 0.001; ****, *p* < 0.0001. CRC: colorectal cancer; Ct: cycle threshold; FDR: false discovery rate; mtDNA-CN: mitochondrial DNA copy number

**Fig. 6:**
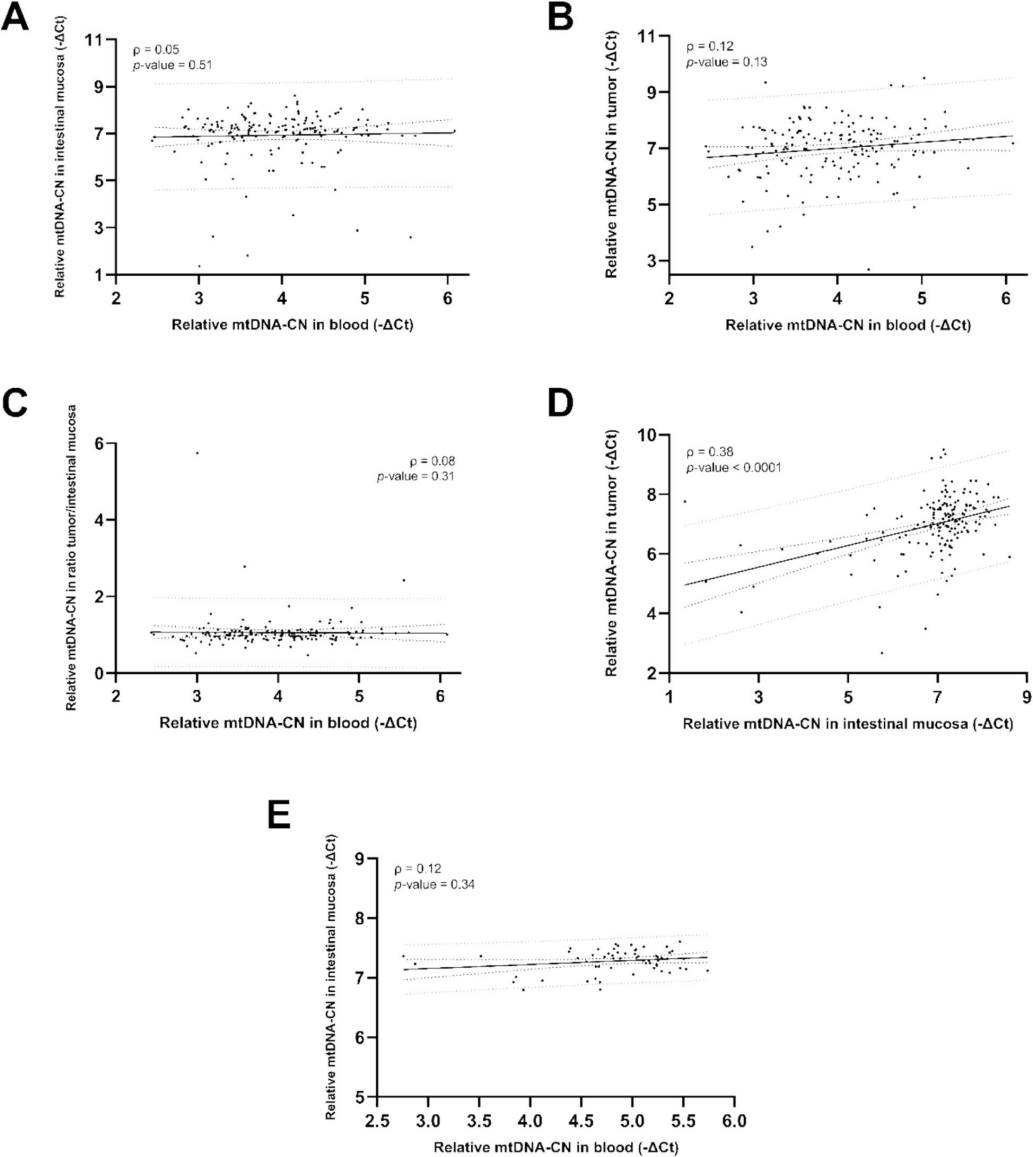
Correlation between relative mtDNA-CNs in tissue types: blood and intestinal mucosa from CRC patients (**A**), blood and tumor from CRC patients (**B**), blood and ratio tumor/intestinal mucosa from CRC patients (**C**), intestinal mucosa and tumor from CRC patients (**D**), and blood and intestinal mucosa from controls (**E**) Data information: Sample sizes of the groups: *n*_blood_ = *n*_intestinal mucosa_ = *n*_tumor_ = *n*_tumor/intestinal mucosa_ = 161 for CRC patients, *n*_blood_ = *n*_intestinal mucosa_ = 61 for controls. In each graph, both Spearman’s rho (ρ) correlation coefficient and the *p*-value are shown, together with the 95% confidence intervals (dark dotted lines) and the 95% prediction intervals (gray dotted lines). CRC: colorectal cancer; Ct: cycle threshold; mtDNA-CN: mitochondrial DNA copy number

A total of 161 CRC patients were included in this analysis (Fig. 2). Firstly, the quantification of relative mtDNA-CN performed by both methods -TaqMan probes and SYBR Green- was shown to be positively, moderately, and significantly correlated in all tissues from CRC patients (Fig. 4). Then, the relative mtDNA-CN was significantly dependent on tissue type (*p* < 0.0001): it was significantly lower in blood from controls when compared to any other solid tissue from controls or CRC patients and was also significantly lower in blood from CRC patients when compared to blood and intestinal mucosa from controls and intestinal mucosa and tumor from CRC patients (Fig. 5). There was a moderate, significant positive correlation between relative mtDNA-CNs in intestinal mucosa and tumors from CRC patients (Fig. 6).

When including the age of the CRC patients in the analysis, there were significant correlations between relative mtDNA-CN and age in specific subgroups of CRC patients: a weak but statistically significant correlation was found between the biomarker in blood and age in patients aged over 55 years, and a significant correlation existed between relative mtDNA-CN in non-tumor intestinal mucosa and age across the full cohort and within the subgroup over 55 years (Additional Results and Supplementary Fig. 3, Additional File 1). Furthermore, the relative mtDNA-CN in individual tissues, as well as the ratio tumor/intestinal mucosa from CRC patients, were compared according to the baseline sociodemographic and clinicopathological characteristics (Additional Results and Supplementary Fig. 4, Additional File 1).

### Relative telomere length: Tissue-specific patterns and correlations

**Fig. 7:**
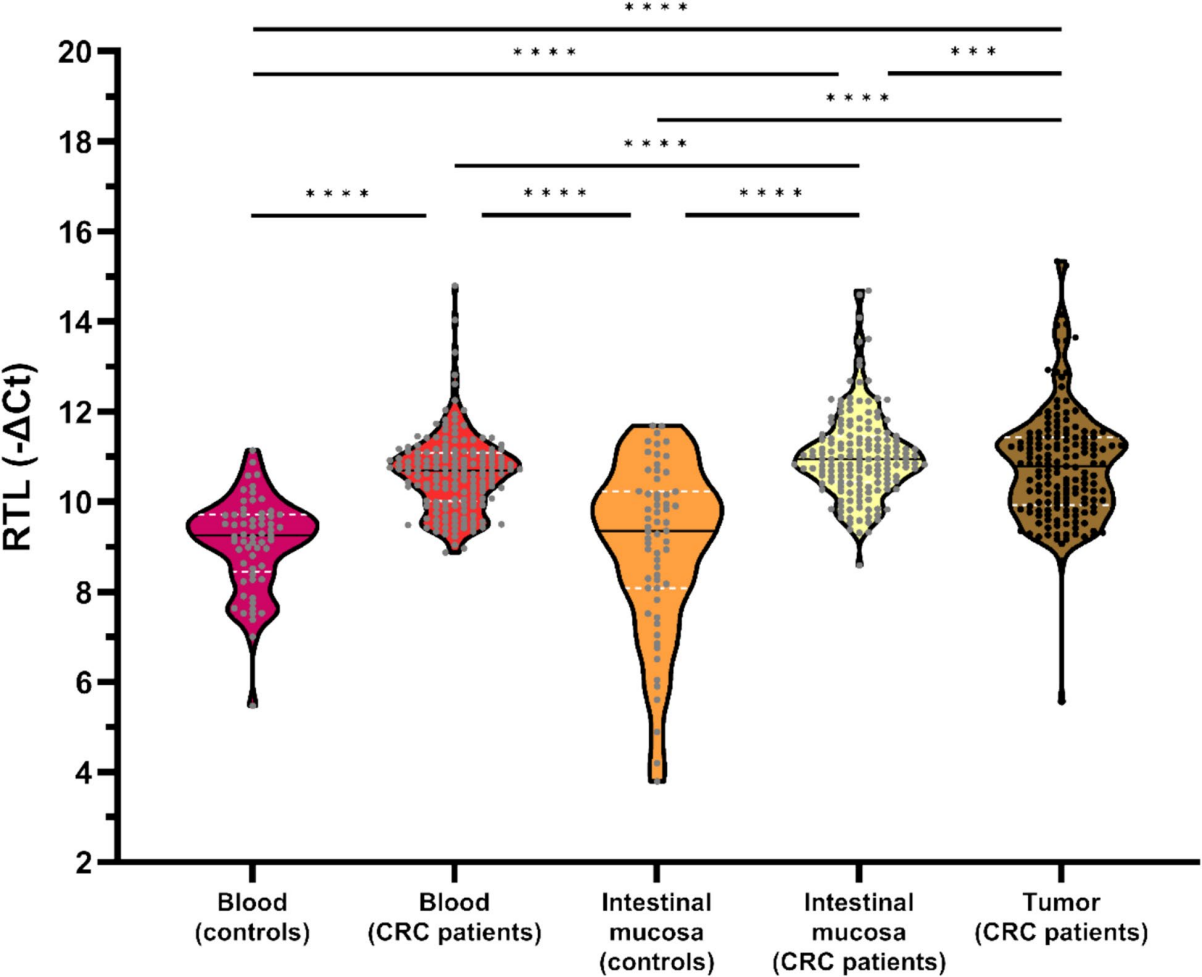
RTL according to tissue type Data information: Sample sizes of the groups: *n*_blood_ = *n*_intestinal mucosa_ = *n*_tumor_ = 161 for CRC patients, *n*_blood_ = *n*_intestinal mucosa_ = 61 for controls. Statistical differences were assessed with the Friedman test followed by pairwise multiple comparisons correcting the resultant *p*-values with the method of Benjamini and Hochberg controlling the FDR at 5% to compare the blood, intestinal mucosa, and tumor groups from CRC patients; with the Kruskal–Wallis test followed by the same multiple comparisons to compare the mean ranks of the blood, intestinal mucosa, and tumor groups from CRC patients to the mean rank of the blood and intestinal mucosa groups from controls as control groups; and with the paired Student *t-*test to compare the blood and intestinal mucosa groups from controls. ***, *p* < 0.001; ****, *p* < 0.0001. CRC: colorectal cancer; Ct: cycle threshold; FDR: false discovery rate; RTL: relative telomere length

**Fig. 8:**
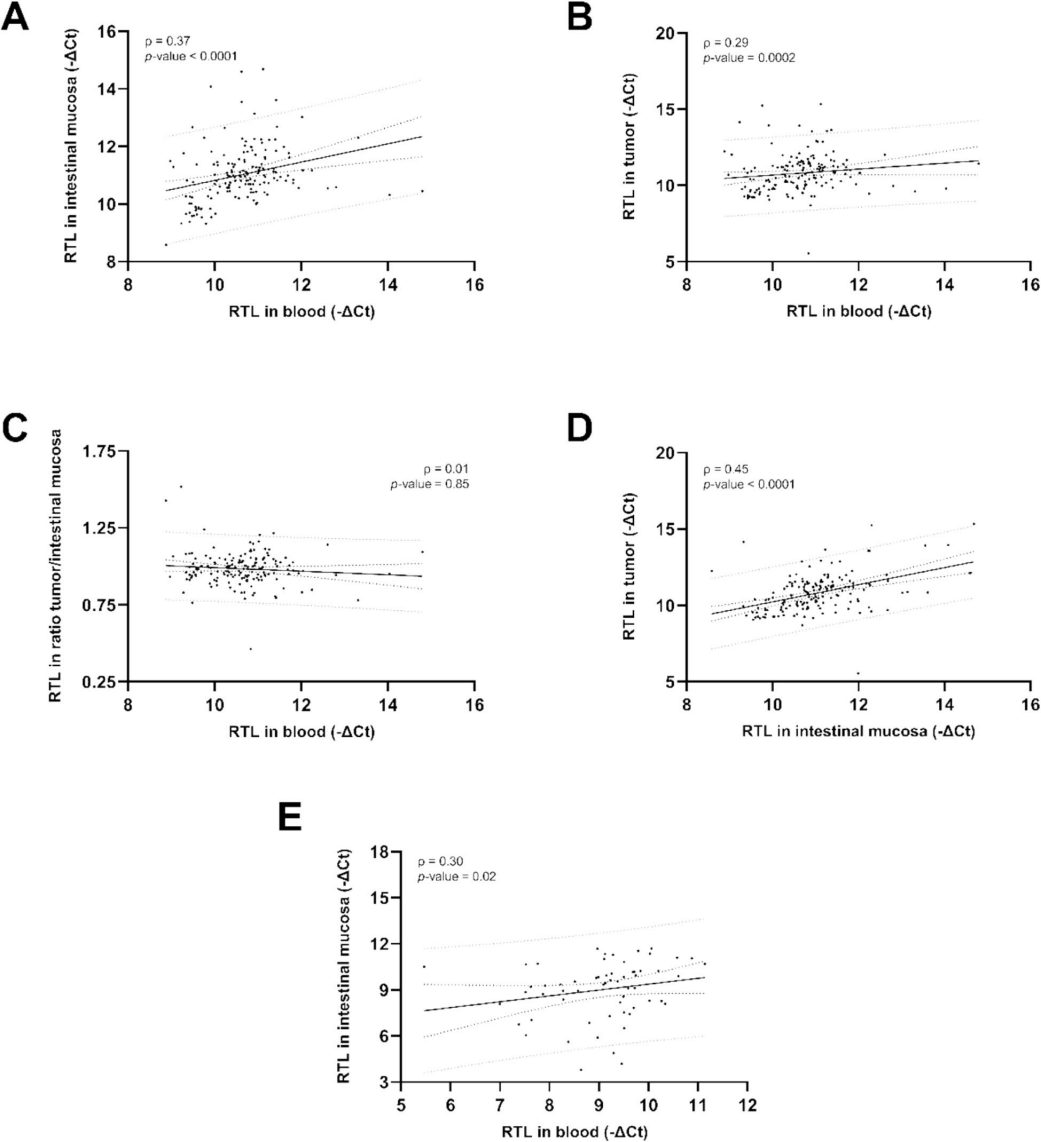
Correlation between RTLs in tissue types: blood and intestinal mucosa from CRC patients (**A**), blood and tumor from CRC patients (**B**), blood and ratio tumor/intestinal mucosa from CRC patients (**C**), intestinal mucosa and tumor from CRC patients (**D**), and blood and intestinal mucosa from controls (**E**) Data information: Sample sizes of the groups: *n*_blood_ = *n*_intestinal mucosa_ = *n*_tumor_ = *n*_tumor/intestinal mucosa_ = 161 for CRC patients, *n*_blood_ = *n*_intestinal mucosa_ = 61 for controls. In each graph, both Spearman’s rho (ρ) correlation coefficient and the *p*-value are shown, together with the 95% confidence intervals (dark dotted lines) and the 95% prediction intervals (gray dotted lines). CRC: colorectal cancer; Ct: cycle threshold; RTL: relative telomere length

A total of 161 CRC patients were included in this analysis (Fig. 2). Firstly, the RTL was shown to be significantly dependent on tissue type (*p* < 0.0001): it was significantly longer in blood from CRC patients than in blood from controls, it was significantly shorter in blood from controls when compared to any solid tissue from CRC patients, it was significantly longer in intestinal mucosa than in tumors from CRC patients, and was also significantly shorter in intestinal mucosa from controls than in any tissue from CRC patients (Fig. 7). There were moderate, significant positive correlations between RTLs in blood and intestinal mucosa, blood and tumor, intestinal mucosa and tumor from CRC patients, and between RTLs in blood and intestinal mucosa from controls (Fig. 8).

When including the age of the CRC patients in the analysis, there were significant correlations between RTL and age in specific subgroups of CRC patients: a significant negative correlation was found between the biomarker in non-tumor intestinal mucosa and age in patients aged over 55 years, and a significant correlation existed between RTL in tumor and age across the full cohort and within the subgroup over 55 years (Additional Results and Supplementary Fig. 5, Additional File 1). Moreover, the RTL in individual tissues, as well as the ratio tumor/intestinal mucosa from CRC patients, were also compared according to the baseline sociodemographic and clinicopathological characteristics (Additional Results and Supplementary Fig. 6, Additional File 1). When stratifying patients by TNM staging, the RTL in blood was significantly longer in TNM III + IV compared to TNM I + II. When stratifying patients by the presence of distant metastasis, the RTL in blood was significantly longer in patients with than in those without metastasis. Finally, when stratifying patients by tumor location, RTL in non-tumor intestinal mucosa was significantly longer in patients with right-sided colon cancer compared to those with rectal cancer.

## Discussion

In this prospective cohort study, we measured relative mtDNA-CN and RTL in samples from 166 sporadic CRC patients. These biomarkers were assessed in blood, tumor-adjacent intestinal mucosa, and tumor biopsies, and their interrelationships were investigated. Biomarker measurements were also compared with those from blood and intestinal mucosa samples from 61 healthy controls without polyps or neoplastic lesions. We also examined these biomarkers in the context of sociodemographic and clinicopathological variables. Finally, survival and multivariable analyses explored their association with the risk of recurrence and mortality.

TL shortening and mitochondrial dysfunction have traditionally been considered hallmarks of aging (Yan et al. 2024; Maldonado et al. 2023; Zhu et al. 2019) and studied as independent contributors to age-related diseases (Borghini et al. 2024). However, given the growing evidence linking these two processes (Zole and Ranka 2018), recent research has focused on their combined role in disease pathogenesis. Consequently, the so-called telomere-mitochondrial axis has been implicated in several pathologies, including cardiovascular (Vostatek et al. 2023; Vecoli et al. 2019; Sabatino et al. 2013), neurological (Koyuncu et al. 2024; Ortega-Vázquez et al. 2023; López-Armas et al. 2023), psychiatric (Ochi et al. 2023; Durand et al. 2022; Cai et al. 2015), metabolic (Utyro et al. 2020), and obstetric diseases (Workalemahu et al. 2016), as well as in exposure to environmental toxins (Wang et al. 2024; Lu et al. 2023; Zhou et al. 2021; Ghosh et al. 2020) and cancer (Lee et al. 2018).

So far, only one study on mtDNA-CN and TL in CRC has been published by Lee et al. (Lee et al. 2018), who analyzed 109 CRC patients and 92 patients with precancerous lesions. Their study consisted of retrospectively collecting formalin-fixed paraffin-embedded tissue samples (tumor and adjacent non-tumor intestinal mucosa) from patients treated at their institution. Relative mtDNA-CN and RTL were quantified using SYBR Green, with cytochrome c oxidase I as the mitochondrial gene and β-globin as the housekeeping gene, and KRAS and BRAF mutations were assessed by pyrosequencing. Most analyses were based solely on tumor tissue data, and no associations were observed with sociodemographic or clinicopathological characteristics, except for increased vascular invasion in tumors with higher relative mtDNA-CN. Survival analyses were limited to OS and performed using Kaplan–Meier curves, yielding no significant associations. No multivariable analyses were conducted, and the retrospective design may have introduced limitations related to data completeness and potential information bias.

Our study is distinct and novel as it includes blood samples from CRC patients as well as blood and intestinal mucosa from healthy individuals, which Lee et al. did not analyze. Given that blood collection is considerably less invasive than colonic biopsies, blood-based analysis presents a promising approach for identifying accessible biomarkers. Our study revealed no correlation between relative mtDNA-CN and RTL in blood from CRC patients, but a significant negative correlation in non-tumor intestinal mucosa and tumors. When stratified by TNM stage, we observed an unexpected trend: in non-tumor intestinal mucosa, the correlation was stronger and significant in TNM I patients, it weakened in TNM II patients and finally became non-significant in TNM III and IV patients. A similar pattern was observed in tumor tissue and the tumor/intestinal mucosa ratio. In contrast, Lee et al. reported positive correlations in both tissues (Lee et al. 2018). However, direct comparison is limited by several differences between the two studies. Firstly, there was a different abundance of rectal cancers and different TNM staging (62% in their cohort versus 32% in ours, and 64% of their patients were TNM III/IV, compared to 45% in our study). On the one hand, unlike us, they did not analyze the effect of neoadjuvant therapy, which many patients likely received for rectal cancer. Their results could have been influenced by this treatment. On the other hand, given that the loss of correlations occurred in advanced stages, the difference in TNM distribution and the lack of TNM stratification in Lee et al.’s analysis may also explain the discrepancy. Furthermore, their samples were microdissected from the same paraffin blocks, with tumor and mucosa located only millimeters apart. In contrast, our fresh-frozen surgical samples ensured a minimum of 5–10 cm between tumor and non-tumor mucosa, reducing potential fixation-related artifacts in, e.g., RT-qPCR analyses. These methodological differences likely contribute to the divergent findings.

To date, only one study has investigated the mtDNA-CN–RTL association in healthy individuals. Tyrka et al. (Tyrka et al. 2015) described a weak but significant correlation between the two biomarkers in blood from 392 adults, independent of age, smoking, and BMI. Our study is the first to examine this association in both blood and intestinal mucosa from healthy individuals. We narrowly missed finding a significant correlation in blood from controls, perhaps due to our much smaller sample size, as obtaining colonic biopsies from healthy individuals without polyps or other suspicious lesions is challenging, especially in older populations. However, we were able to demonstrate a significant negative correlation in the intestinal mucosa from these participants. The magnitude of this association was virtually identical to that observed in the intestinal mucosa of CRC patients. This supports the aforementioned hypothesis that disease progression may disrupt crosstalk homeostasis between the two processes.

In summary, our findings indicate a loss of correlation between mtDNA-CN and RTL with advancing TNM stage. Although our study does not include mechanistic analyses of mitochondria- or telomere-related signaling pathways, the observed tissue- and stage-dependent correlations between mtDNA-CN and RTL provide a phenotypic demonstration of the telomere–mitochondrial axis in human CRC. The loss of this correlation with advancing stage supports the hypothesis that tumor progression disrupts the coordination between mitochondrial function and telomeric maintenance, consistent with findings in other malignancies such as intestinal gastric cancer (Jung et al. 2017).

The loss of correlation between mtDNA-CN and RTL with advancing TNM stage likely reflects a progressive disruption of cellular homeostasis between telomeric maintenance and mitochondrial biogenesis during tumor evolution. In early-stage tumors, mitochondrial and telomeric integrity may still be under partial control of shared regulatory networks, such as p53 signaling, TERT translocation to mitochondria, and PGC-1α/NRF1-mediated mitochondrial biogenesis pathways (Sahin et al. 2011; Schank et al. 2020), which together coordinate oxidative stress responses (Marinaccio et al. 2023; Sahin et al. 2011). As tumors progress, increasing oxidative and metabolic stress, genomic instability, and altered redox signaling can promote both telomere attrition and mitochondrial dysfunction (Kudryavtseva et al. 2016); however, these processes become increasingly dysregulated and independent. Advanced tumors frequently exhibit metabolic reprogramming (e.g., the Warburg effect), mtDNA mutations, and aberrant mitophagy (Zhang et al. 2025; Li et al. 2025), which may further decouple mitochondrial quantity from telomere status. Consequently, the disappearance of the mtDNA-CN–RTL association across TNM stages could represent a phenotypic signature of this breakdown in telomere–mitochondrial crosstalk accompanying malignant progression.

This interpretation aligns with our observation that the negative correlation between mtDNA-CN and RTL was strongest in TNM I and progressively weakened in more advanced stages. Together, these results suggest that the telomere-mitochondrial axis remains relatively intact in early tumorigenesis but becomes increasingly deregulated as cancer cells acquire metabolic and genomic adaptations that support uncontrolled proliferation.

This study is also the first to investigate the relationship between relative mtDNA-CN in blood and recurrence risk in CRC patients. Notably, higher relative mtDNA-CN in blood was associated with a lower risk of disease recurrence. This association remained statistically significant after adjusting for other prognostic factors. We did not observe the same trend in tumor tissue, perhaps due to tumor heterogeneity in our sample. To date, only one study by Chen et al. (Chen et al. 2024a) had already reported high mtDNA-CN as an independent protective factor for recurrence, but their analysis was limited to tumor tissue from MSI-high CRC patients, who generally have a more favorable prognosis.

Our findings thus indicate that blood-based mtDNA-CN may help to identify patients at higher risk of recurrence, especially during the first two years after diagnosis, when 84% of recurrences occurred in our study. In this way, blood mtDNA-CN could represent a valuable complement to established prognostic factors described in the literature and used in clinical practice, such as postoperative positivity for circulating tumor DNA and the persistence of elevated tumor markers, such as carbohydrate antigen 19–9 (CA19-9) or carcinoembryonic antigen (CEA) (Wood et al. 2025; Chen et al. 2022; Ryuk et al. 2014). All these factors, easily obtainable through routine blood tests, may serve as potential non-invasive monitoring tools to identify patients at increased risk and offer them closer postoperative follow-up to detect recurrences early.

Notably, the absence of a correlation between both biomarkers in blood does not contradict the prognostic value of blood mtDNA-CN. Leukocyte mtDNA-CN and leukocyte RTL reflect distinct systemic processes (hematopoietic dynamics, inflammation, oxidative stress, and mitochondrial biogenesis) and therefore are not required to covary. In contrast, tumor tissue and adjacent mucosa capture local metabolism and axis behavior that appear to be stage dependent. These differences across tissues and stages are biologically expected as they reflect distinct contexts. The independent association between higher blood mtDNA-CN at diagnosis and lower relapse risk, sustained in multivariable models, supports blood mtDNA-CN as a clinically practical, minimally invasive biomarker candidate for risk stratification in CRC patients.

TaqMan probes and SYBR Green are common RT-qPCR methods. TaqMan offers higher specificity through sequence-specific probes and allows multiplexing (Navarro et al. 2015; Matsenko et al. 2008), while SYBR Green is cheaper and faster but less specific, as it binds to any amplified double-stranded DNA (dsDNA) (Navarro et al. 2015; Shahrajabian and Sun 2024). Although TaqMan is more sensitive and specific, several studies suggest that both methods can offer comparable results depending on the target sequence (Peris et al. 2021; Soltany-Rezaee-Rad et al. 2015; Tajadini et al. 2014; Cao and Shockey 2012). Ours is the first study directly comparing these two approaches for mtDNA-CN quantification, showing moderate positive correlations across all tissue types. Given that mtDNA-CN is not a low-abundance target -with hundreds to thousands of copies per cell (Wang et al. 2006)- and thus the analysis does not require high sensitivity, our findings support the comparability of both methods for this application.

Recent literature demonstrates the growing evidence of mitochondrial dysfunction as a critical factor in disease pathogenesis (Zong et al. 2024; Suzuki et al. 2024; Dong et al. 2023; Chan 2020), as it contributes to (or may be a consequence of) increased reactive oxygen species (ROS) production and other pathological processes (Liu et al. 2024, 2022; Alula et al. 2023; Zhai et al. 2022; Zhao et al. 2021). Impaired mitochondrial function can interrupt the nuclear-mitochondrial communication network, a dynamic process whose dysregulation has been linked to cancer (Palmer et al. 2021). mtDNA-CN has been identified as a marker of mitochondrial dysfunction, making it a valuable tool for investigating its role in diseases (Xu et al. 2024; Chiang et al. 2020).

Despite its potential, few studies have explored mtDNA-CN in CRC. Only eight have analyzed tissue samples (Lee et al. 2018; Chen et al. 2024a, 2024b; Zhu et al. 2022; Kang et al. 2022; Zhao et al. 2022; Osch et al. 2015; Potenza et al. 2011) and four, blood samples (Guan et al. 2023; Yang et al. 2020, 2019; Huang et al. 2014). To our knowledge, this is the first to examine mtDNA-CN in both circulating cells and solid tissues in CRC, allowing for a more comprehensive understanding of its role. We found significantly higher mtDNA-CN in all solid tissues compared to blood, regardless of disease status, reflecting the higher metabolic activity of colonic tissue, which demands increased energy production and higher mtDNA-CN (Chodowiec et al. 2024; Stojanović et al. 2022). In CRC patients, this suggests preserved mitochondrial dynamics in non-blood compartments. Although mtDNA-CN levels did not differ significantly between tumor and intestinal mucosa, a downward trend was observed. This may reflect the metabolic shift in tumors from oxidative phosphorylation to anaerobic glycolysis (the Warburg effect), reducing mitochondrial demand and leading to decreased mtDNA-CN (Kim and Dang 2006; Meierhofer 2004). Our findings support this hypothesis and align with previous reports (Chen et al. 2024a), highlighting the need for further investigation.

Telomeres protect chromosome ends from damage (Smith et al. 2020) and their dysfunction occurs in aging and aging-related diseases (Rossiello et al. 2022). Unlike mtDNA-CN, their link to disease, including CRC, is well documented. Although many studies exist, only three (Kroupa et al. 2019; Valls-Bautista et al. 2015; O’Sullivan et al. 2006) have examined TL in both blood and solid tissues from CRC patients, making our study an important addition to this limited body of evidence.

We confirmed significantly shorter RTL in tumor tissue compared to the adjacent mucosa from CRC patients, in line with the literature (Kroupa et al. 2019, 2023; Valls-Bautista et al. 2015; Balc’h et al. 2017; Fernández-Marcelo et al. 2016; Valls et al. 2011; Garcia-Aranda et al. 2006; Gertler et al. 2004). This trend has also been described in precancerous lesions such as adenomas (Riegert-Johnson et al. 2012; Raynaud et al. 2008). Considering all the evidence together, telomere shortening is suggested to be not only an early event in CRC carcinogenesis but one of the initial events in the onset of the disease (Nikolouzakis et al. 2018; Aghagolzadeh and Radpour 2016). Interestingly, tumor RTL was significantly longer than in the blood from controls, but not significantly different from blood in CRC patients, and RTL in blood was significantly higher in CRC patients than in controls. This suggests a systemic effect of the disease – similar to findings in benign prostatic hyperplasia (Zole et al. 2024) and breast cancer (Limardi et al. 2024). We also observed moderate, positive RTL correlations between all tissue types in CRC patients and controls. While the correlation between intestinal mucosa and tumors was reported before (Fernández-Marcelo et al. 2016; Mehrez et al. 2019), this was the first time demonstrating correlations between blood and the two solid tissues.

The limitations of this study include the lack of precancerous lesion samples (e.g., colon adenomas), which prevented a more detailed assessment of biomarker changes along the intestinal mucosa-adenoma-carcinoma sequence. Another limitation is the absence of additional markers, such as TERT or mitochondrial transcription factor A (TFAM) expression, which could have further elucidated the telomere-mitochondrial crosstalk. Additionally, leukocyte subset composition was not directly controlled; future studies should integrate cell-type deconvolution approaches to refine blood-based mtDNA-CN risk estimates. Finally, healthy individuals were younger than CRC patients, and a different proportion of women was also observed between the two groups, which together may have affected the comparison. Future research should address these limitations through prospective studies to further elucidate the role of mtDNA-CN and TL in CRC prognosis and progression. However, the strengths of this study lie in its novelty and comprehensiveness. Unlike most studies, we included healthy controls and analyzed relative mtDNA-CN and RTL in both blood and solid tissues from CRC patients. Also, we investigated novel interactions and prognostic associations, including the first-ever analysis of mtDNA-CN in both circulating cells and solid tissues from CRC patients. Finally, the prospective follow-up enabled systematic data collection and helped mitigate, to some extent, potential information and confounding bias, although residual confounding inherent to observational design must always be acknowledged.

## Conclusions

In this prospective cohort study, we showed a statistically significant negative correlation between relative mtDNA-CN and RTL in intestinal mucosa from healthy individuals, and in intestinal mucosa, tumor tissue, and the tumor-to-intestinal mucosa ratio from CRC patients. This association gradually weakened and eventually disappeared with tumor progression, suggesting dysregulation of the telomere-mitochondrial axis in advanced stages of disease. We also report, for the first time, an association between higher relative mtDNA-CN in blood and lower recurrence risk in CRC. Additionally, the quantification of relative mtDNA-CN performed by both methods, TaqMan probes and SYBR Green, showed a moderate, positive correlation, indicating that these methods may be comparable for this purpose. Furthermore, this study is the first to compare relative mtDNA-CN across both circulating cells and solid tissues, demonstrating consistently higher levels in solid tissues compared to blood in CRC patients. The findings concerning RTL are in line with previously reported data, thereby reinforcing existing knowledge.

Overall, this study provides insights into the largely unexplored telomere-mitochondrial axis in CRC, highlighting its potential role in disease progression and prognosis.

## Supplementary Information

Supplementary Material 1: Additional Materials and Methods and Additional Results, including Supplementary Figures 1-6 and Supplementary Tables 1-13.

## Data Availability

The datasets used and analyzed during the current study are available from the corresponding author on reasonable request.

## Abbreviations

*ALB*: Albumin gene
ANOVA: Analysis of variance
BMI: Body mass index
CA19-9: Carbohydrate antigen 19–9
CEA: Carcinoembryonic antigen
CI: Confidence interval
CN: Copy number
CRC: Colorectal cancer
Ct: Cycle threshold
dsDNA: Double-stranded DNA
EDTA: Ethylenediaminetetraacetic acid
FDR: False discovery rate
HPLC: High-performance liquid chromatography
HR: Hazard ratio
ICD-10: International Statistical Classification of Diseases and Related Health Problems 10^th^ Revision
IQR: Interquartile range
MSI: Microsatellite instability
mtDNA: Mitochondrial DNA
mtDNA-CN: Mitochondrial DNA copy number
*MT-ND1*: NADH-ubiquinone oxidoreductase chain 1 gene
nDNA: Nuclear DNA
OS: Overall survival
PCR: Polymerase chain reaction
PI: Prediction interval
P_50_: Median
RFS: Relapse-free survival
ROS: Reactive oxygen species
RTL: Relative telomere length
RT-qPCR: Real-time quantitative polymerase chain reaction
*s*: Standard deviation
SEM: Standard error of the mean
TERT: Telomerase reverse transcriptase
TFAM: Mitochondrial transcription factor A
TL: Telomere length
TNM: Tumor-Node-Metastasis system
UICC: Union for International Cancer Control
$$\overline{x }$$: Arithmetic mean
ρ: Spearman’s rho correlation coefficient
