# Supplementary material for: Unraveling the telomere-mitochondrial axis in colorectal cancer: Results from a prospectively followed cohort
Source: Mol Med. 2026 Feb 21;32:43. doi: 10.1186/s10020-026-01423-6 (PMC13032530; doi:10.1186/s10020-026-01423-6)
Supplement: Supplementary file 1 — Supplementary Material 1: Additional Materials and Methods and Additional Results, including Supplementary Figures 1-6 and Supplementary Tables 1-13. [file 10020_2026_1423_MOESM1_ESM.docx]

# Unraveling the telomere-mitochondrial axis in colorectal cancer: Results from a prospectively followed cohort

Adrián Gil-Korilis^1,2,#^, Jorge Ergui-Arbizu^1,3,#^, Natálie Danešová^1,4,#^, Petr Hanák^1,#^, Kristýna Tomášová^1,4^, Anna Valíčková^1,5^, Josef Horák^1^, Manuel Gentiluomo^6^, Miroslav Levý^7^, Soňa Křivonosková^8^, Jan Král^9,10^, Jiří Jungwirth^11,12^, Ludmila Vodičková^1,4,5,13^, Veronika Vymetálková^1^, Amaya Azqueta^14^, Daniele Campa^6^, Pavel Vodička^1,4,5,13^, Soňa Vodenková^1,4*^

^#^ These authors share first co-authorship. * Corresponding author.

^1^ Department of Molecular Biology of Cancer, Institute of Experimental Medicine of the Czech Academy of Sciences, Prague, Czech Republic

^2^ School of Medicine, University of Navarra, Pamplona, Spain

^3^ School of Sciences, University of Navarra, Pamplona, Spain

^4^ Biomedical Centre, Faculty of Medicine in Pilsen, Charles University, Pilsen, Czech Republic

^5^ Institute of Biology and Medical Genetics, First Faculty of Medicine, Charles University, Prague, Czech Republic

^6^ Unit of Genetics, Department of Biology, University of Pisa, Pisa, Italy

^7^ Department of Surgery, First Faculty of Medicine, Charles University and Thomayer University Hospital, Prague, Czech Republic

^8^ Department of Oncology, First Faculty of Medicine, Charles University and Thomayer University Hospital, Prague, Czech Republic

^9^ Department of Internal Medicine, Second Faculty of Medicine, Charles University and Motol University Hospital, Prague, Czech Republic

^10^ Department of Hepatogastroenterology, Institute for Clinical and Experimental Medicine, Prague, Czech Republic

^11^ Institute of Physiology, First Faculty of Medicine, Charles University, Prague, Czech Republic

^12^ Department of Gastroenterology, Libera Scientia, Prague, Czech Republic

^13^ Biomedical Centre Martin, Jessenius Faculty of Medicine in Martin, Comenius University in Bratislava, Martin, Slovakia

^14^ Department of Pharmaceutical Sciences, School of Pharmacy and Nutrition, University of Navarra, Pamplona, Spain

**Running title:** Mitochondrial DNA copy number and telomere length in colorectal cancer

**Corresponding author:** Email: sona.vodenkova@iem.cas.cz, Tel: +420 241062251, Full postal address: Department of the Molecular Biology of Cancer, Institute of Experimental Medicine of the Czech Academy of Sciences, Vídeňská 1083, 142 00 Prague, Czech Republic.

# Additional materials and methods

### Supplementary Table 1. STROBE checklist for reporting observational studies^1^.

|  | **Item** | **Recommendation** | **Page** |
| --- | --- | --- | --- |
| **Title and abstract** | 1 | (*a*) Indicate the study’s design with a commonly used term in the title or the abstract | 1-2 |
|  |  | (*b*) Provide in the abstract an informative and balanced summary of what was done and what was found | 1-2 |
| **Introduction** | | | |
| **Background/****rationale** | 2 | Explain the scientific background and rationale for the investigation being reported | 2-3 |
| **Objectives** | 3 | State specific objectives, including any prespecified hypotheses | 3 |
| **Methods** | | | |
| **Study design** | 4 | Present key elements of study design early in the paper | 3 |
| **Setting** | 5 | Describe the setting, locations, and relevant dates, including periods of recruitment, exposure, follow-up, and data collection | 3-4 |
| **Participants** | 6 | (*a*) *Cohort study*—Give the eligibility criteria, and the sources and methods of selection of participants. Describe methods of follow-up  *Case-control study*—Give the eligibility criteria, and the sources and methods of case ascertainment and control selection. Give the rationale for the choice of cases and controls  *Cross-sectional study*—Give the eligibility criteria, and the sources and methods of selection of participants | 3-4 |
|  |  | (*b*) *Cohort study*—For matched studies, give matching criteria and number of exposed and unexposed  *Case-control study*—For matched studies, give matching criteria and the number of controls per case | NA |
| **Variables** | 7 | Clearly define all outcomes, exposures, predictors, potential confounders, and effect modifiers. Give diagnostic criteria, if applicable | 6-7 |
| **Data sources/** **measurement** | 8 | For each variable of interest, give sources of data and details of methods of assessment (measurement). Describe comparability of assessment methods if there is more than one group | 4-6 |
| **Bias** | 9 | Describe any efforts to address potential sources of bias | 4 |
| **Study size** | 10 | Explain how the study size was arrived at | 6 |
| **Quantitative** **variables** | 11 | Explain how quantitative variables were handled in the analyses. If applicable, describe which groupings were chosen and why | 6-7 |
| **Statistical** **methods** | 12 | (*a*) Describe all statistical methods, including those used to control for confounding | 6-7 |
|  |  | (*b*) Describe any methods used to examine subgroups and interactions | 6-7 |
|  |  | (*c*) Explain how missing data were addressed | 7 |
|  |  | (*d*) *Cohort study*—If applicable, explain how loss to follow-up was addressed  *Case-control study*—If applicable, explain how matching of cases and controls was addressed  *Cross-sectional study*—If applicable, describe analytical methods taking account of sampling strategy | 6-7 |
|  |  | (*e*) Describe any sensitivity analyses | NA |
| **Results** | | | |
| **Participants** | 13 | (a) Report numbers of individuals at each stage of study—eg numbers potentially eligible, examined for eligibility, confirmed eligible, included in the study, completing follow-up, and analysed | 7 |
|  |  | (b) Give reasons for non-participation at each stage | 7 |
|  |  | (c) Consider use of a flow diagram | 8 |
| **Descriptive** **data** | 14 | (a) Give characteristics of study participants (eg demographic, clinical, social) and information on exposures and potential confounders | 9 |
|  |  | (b) Indicate number of participants with missing data for each variable of interest | NA |
|  |  | (c) *Cohort study*—Summarise follow-up time (eg, average and total amount) | 7 |
| **Outcome data** | 15 | *Cohort study*—Report numbers of outcome events or summary measures over time | 7 |
|  |  | *Case-control study—*Report numbers in each exposure category, or summary measures of exposure | NA |
|  |  | *Cross-sectional study—*Report numbers of outcome events or summary measures | NA |
| **Main results** | 16 | (*a*) Give unadjusted estimates and, if applicable, confounder-adjusted estimates and their precision (eg, 95% confidence interval). Make clear which confounders were adjusted for and why they were included | 12 |
|  |  | (*b*) Report category boundaries when continuous variables were categorized | 12 |
|  |  | (*c*) If relevant, consider translating estimates of relative risk into absolute risk for a meaningful time period | NA |
| **Other analyses** | 17 | Report other analyses done—eg analyses of subgroups and interactions, and sensitivity analyses | Suppl 16 |
| **Discussion** | | | |
| **Key results** | 18 | Summarise key results with reference to study objectives | 11-12 |
| **Limitations** | 19 | Discuss limitations of the study, taking into account sources of potential bias or imprecision. Discuss both direction and magnitude of any potential bias | 19 |
| **Interpretation** | 20 | Give a cautious overall interpretation of results considering objectives, limitations, multiplicity of analyses, results from similar studies, and other relevant evidence | 24-31 |
| **Generalisability** | 21 | Discuss the generalisability (external validity) of the study results | 11-19 |
| **Other information** | | | |
| **Funding** | 22 | Give the source of funding and the role of the funders for the present study and, if applicable, for the original study on which the present article is based | 20 |

## Measurement of relative mtDNA copy number

The relative mtDNA-CN was determined by real-time quantitative polymerase chain reaction (RT-qPCR) amplification in a QuantStudio™ 6 Flex Real-Time PCR System (Applied Biosystems^®^, Thermo Fisher Scientific, Waltham, USA). The results were analyzed with QuantStudio™ Real-Time PCR Software. To assess this determination, the mitochondria-specific gene NADH-ubiquinone oxidoreductase chain 1 (*MT-ND1*) was amplified and compared to the nuclear single-copy gene albumin (*ALB*) as the housekeeping gene.

A duplex TaqMan probe-based approach (Applied Biosystems^®^, Thermo Fisher Scientific) was utilized, allowing for a parallel measurement of both target genes. The *MT-ND1* probe was labeled with a VIC reporter dye, while the *ALB* probe was labeled with a FAM reporter dye. Prior to measurements, template DNA from each sample was adjusted to a concentration of 5 ng/μL with UltraPure™ DNase/RNase-Free Distilled Water (Invitrogen™, Thermo Fisher Scientific, Waltham, USA). All samples were pipetted in triplicate by an OT-2 Pipetting Robot (Opentrons, New York City, USA). Reactions were run in a 384-well optical plate (MicroAmp™ Optical 384-Well Reaction Plate, Applied Biosystems^®^, Thermo Fisher Scientific).

The RT-qPCR mixture of 10 μL per well was prepared as follows: 5 μL TaqMan™ Universal Master Mix II, no UNG (Applied Biosystems^®^, Thermo Fisher Scientific), 3 μL UltraPure™ DNase/RNase-Free Distilled Water, 0.5 μL *MT-ND1* primer and probe (Assay ID Hs02596873_s1, TaqMan^®^ Gene Expression Assays, Applied Biosystems^®^, Thermo Fisher Scientific), 0.5 μL *ALB* primer and probe (Assay ID Hs05910690_cn, TaqMan^®^ Copy Number Assays, Applied Biosystems^®^, Thermo Fisher Scientific), and 1 μL of template DNA. The thermal cycling protocol consisted of an initial denaturation at 95 °C for 10 min, followed by 40 cycles composed of a denaturation at 95 °C for 15 sec and elongation at 60 °C for 1 min. The fluorescence signal was measured at the end of each cycle at 60 °C.

The cycle threshold (Ct) value was determined by the software employed. The results were expressed as relative quantification by calculating the ΔCt for each sample, being ΔCt*_sample_* = Ct*_MT-ND1_* – Ct*_ALB_*. The sample was excluded if the ΔCt standard deviation of the triplicate was *s* ≥ 0.4.

Furthermore, relative mtDNA-CN was also quantified with an alternative method based on a monochromatic duplex RT-qPCR approach employing SYBR Green, as previously described^2^, to compare it with the method using TaqMan probes. Thus, the determination was conducted by RT-qPCR amplification in a ViiA™ 7 Real-Time PCR System (Applied Biosystems^®^, Thermo Fisher Scientific), and the results were analyzed with QuantStudio™ Real-Time PCR Software. The *MT-ND1* gene was again used as the target and compared to the *ALB* gene as the housekeeping gene. This approach involved a monochromatic duplex RT-qPCR, measuring both target and reference genes together in a single reaction, in two sequential blocks of RT-qPCR, head to tail one to the other. SYBR Green-based SYTO™ 9 green fluorescent dye (Invitrogen™, Thermo Fisher Scientific) was used for the detection of both amplification products. Primer sequences are listed in **Supplementary Table 2, Additional File 1**. *ALB* primers were constructed with a GC-clamp at the 5′ end to increase the melting temperature^3^. Amplifications were performed in triplicate on 384-well optical plates.

The RT-qPCR mixture per well consisted of 10 μL, including 8 μL water, 2 μL 5x HOT FIREPol^®^ Probe qPCR Mix Plus (ROX) (Solis BioDyne, Tartu, Estonia), 1.5 μmol/L SYTO™ 9 green fluorescent nucleic acid dye (Invitrogen™, Thermo Fisher Scientific), and 5 ng of template DNA. The thermal cycling protocol consisted of an initial denaturation at 95 °C for 5 min, followed by two preliminary cycles at 95 °C for 15 sec and 60 °C for 1 min, a first amplification block for *MT-ND1* of 30 cycles composed of a denaturation step at 85 °C for 15 sec and an elongation step at 65 °C for 1 min, and a second amplification block for *ALB* of 35 cycles composed of a denaturation step at 95 °C for 15 sec and two elongation steps at 85 °C for 30 sec and 84 °C for 30 sec. The fluorescence signal was measured at the end of each cycle at 65 °C for *MT-ND1* and 84 °C for *ALB*. Following amplification, a melting curve analysis was performed to verify the specificity of the products. This consisted of an initial step at 95 °C for 15 sec, another step at 60 °C for 1 min, and a final stage at 95 °C for 15 sec with a temperature gradient of 0.05 °C/sec from 60 to 95 °C.

The Ct value was determined by the software used. The sample was excluded if the individual Ct values deviated from the triplicate average by more than 5% of the standard deviation. Relative mtDNA-CN was expressed as the ratio Ct*_MT-ND1_*/Ct*_ALB_* according to the Pfaffl method^4^.

## Measurement of relative telomere length

The RTL of the samples was determined by RT-qPCR amplification in a QuantStudio™ 6 Flex Real-Time PCR System (Applied Biosystems^®^, Thermo Fisher Scientific, Waltham, USA). The results were analyzed with QuantStudio™ Real-Time PCR Software. Canonical 5’-TTAGGG-3’ telomere motif repeats were amplified and compared to the nuclear single-copy gene albumin (*ALB*) as the housekeeping gene.

A monochromatic duplex RT-qPCR approach was employed, measuring both target and reference genes together in a single reaction in two sequential blocks of RT-qPCR, head to tail one to the other. SYBR Green-based SYTO™ 9 green fluorescent dye (Invitrogen™, Thermo Fisher Scientific) was used for the detection of both amplification products. High-performance liquid chromatography (HPLC) primers for telomere motif were obtained from Generi Biotech (Hradec Králové, Czech Republic), and standard purified primers for *ALB* were obtained from Merck KGaA (Sigma-Aldrich, Darmstadt, Germany) (primer sequences are listed in **Supplementary Table 2, Additional File 1**). *ALB* primers were constructed with a GC-clamp at the 5′ end to increase the melting temperature^3^. Before the measurements, template DNA from each sample was adjusted to a concentration of 1 ng/μL with UltraPure™ DNase/RNase-Free Distilled Water (Invitrogen™, Thermo Fisher Scientific, Waltham, USA). Samples were pipetted in triplicate by an OT-2 Pipetting Robot into a 384-well optical plate (MicroAmp™ Optical 384-Well Reaction Plate with Barcode, Applied Biosystems^®^, Thermo Fisher Scientific).

The RT-qPCR mixture per well consisted of 10.72 μL, including 6.61 μL UltraPure™ DNase/RNase-Free Distilled Water, 2.16 μL 5x HOT FIREPol^®^ Probe qPCR Mix Plus (ROX) (Solis BioDyne, Tartu, Estonia), 0.02 μL telomere motif forward primer (100 μM, final concentration 0.19 μM), 0.04 μL telomere motif reverse primer (100 μM, final concentration 0.37 μM), 0.02 μL *ALB* forward primer (100 μM, final concentration 0.19 μM), 0.04 μL *ALB* reverse primer (100 μM, final concentration 0.37 μM), 0.02 μL SYTO™ 9 green fluorescent nucleic acid dye (Invitrogen™, Thermo Fisher Scientific), and 1.81 μL of template DNA. The thermal cycling protocol consisted of an initial denaturation at 95 °C for 15 min, followed by two preliminary cycles at 95 °C for 20 sec and 49 °C for 1 min (needed to create the telomere motif forward primer extension product to serve as the template for the telomere motif reverse primer, as proposed by Cawthon, 2009 ^3^), a first amplification block for telomere motif of 25 cycles composed of a denaturation step at 85 °C for 20 sec and an elongation step at 59 °C for 30 sec, and a second amplification block for *ALB* of 33 cycles composed of a denaturation step at 95 °C for 15 sec and two elongation steps at 85 °C for 30 sec and 84 °C for 30 sec. The fluorescence signal was measured at the end of each cycle at 59 °C for the telomere motif and 84 °C for *ALB*. Following amplification, a melting curve analysis was performed to verify the specificity of the products. This consisted of an initial step at 95 °C for 15 sec, another step at 60 °C for 1 min, and a final stage at 95 °C for 15 sec with a temperature gradient of 0.05 °C/sec from 60 to 95 °C.

The Ct value was determined by the software employed. The results were expressed as relative quantification by calculating the ΔCt for each sample, being ΔCt*_sample_* = Ct*_telomere_* – Ct*_ALB_*. The sample was excluded if the ΔCt standard deviation of the triplicate was *s* ≥ 0.4.

### Supplementary Table 2. Sequences of the primers used in this study.

| **Primer** | **Sequence (5’-3’)^a,b^** | **Source** |
| --- | --- | --- |
| *ALB* forward | CGGCGGCGGGCGGCGCGGGCTGGGCGGCCATGCT  TTTCAGCTCTGCAAGTC | ^2^ |
| *ALB* reverse | GCCCGGCCCGCCGCGCCCGTCCCGCCGAGCATTAA  GCTCTTTGGCAACGTAGGTTTC | ^2^ |
| *MT-ND1* forward | CCCTAAAACCCGCCACATCT | ^2^ |
| *MT-ND1* reverse | GAGCGATGGTGAGAGCTAAGGT | ^2^ |
| Telomere motif forward | ACACTAAGGTTTGGGTTTGGGTTTGGGTTTGGGTT  AGTGT | ^3^ |
| Telomere motif reverse | TGTTAGGTATCCCTATCCCTATCCCTATCCCTATCCC  TAACA | ^3^ |

^a^ The GC-clamp added to the 5′ ends of *ALB* primers to raise the melting temperature is underlined. ^b^ The primers used for the measurement of relative mtDNA-CN using TaqMan probes are not listed here. *ALB:* albumin; *MT-ND1*: NADH-ubiquinone oxidoreductase chain 1.

# Additional results

## Survival analysis

A total of 159 CRC patients were included in this analysis when employing relative mtDNA-CN, as two patients were excluded due to unclear dates, as shown in **Figure 2.** When dividing the CRC patients into two groups according to their relative mtDNA-CN in blood (< P_50_, *n* = 79; > P_50_, *n* = 80), relapse occurred in 20 patients (25.32%) in the < P_50_ group and in 11 (13.75%) in the > P_50_ group (HR = 0.48, 95% CI 0.24-0.97; ***p* = 0.04**), whereas 15 patients (18.99%) died in the < P_50_ group and 11 (13.75%) in the > P_50_ group (HR = 0.63, 95% CI 0.29-1.37; *p* = 0.25), as shown in **Supplementary Figure 1.** In the < P_50_ group, relapse-free survival (RFS) was 78.6% (95% CI 66.4-86.8) at 1.5 years and 55.8% (95% CI 34.9-72.4) at 3 years, and overall survival (OS) was 88.0% (95% CI 77.3-93.8) at 1.5 years and 66.5% (95% CI 46.9-80.2) at 3 years, as shown in **Supplementary Table 2.** In the > P_50_ group, RFS was 89.2% (95% CI 78.6-94.7) at 1.5 years and 78.1% (95% CI 62.3-87.9) at 3 years, and OS was 92.0% (95% CI 83.1-96.3) at 1.5 years and 80.1% (95% CI 65.7-89.0) at 3 years. No median survival times for RFS nor OS were reached in either group.

When dividing the CRC patients into two groups according to their relative mtDNA-CN in non-tumor intestinal mucosa (< P_50_, *n* = 79; > P_50_, *n* = 80), relapse occurred in 13 patients (16.46%) in the < P_50_ group and in 18 (22.50%) in the > P_50_ group (HR = 1.36, 95% CI: 0.67-2.74; *p* = 0.40), whereas 12 patients (15.19%) died in the < P_50_ group and 14 (17.50%) in the > P_50_ group (HR = 1.06, 95% CI: 0.49-2.28; *p* = 0.89), as shown in **Supplementary Figure 1.** In the < P_50_ group, RFS was 80.5% (95% CI: 68.0-88.5) at 1.5 years and 76.7% (95% CI: 61.9-86.3) at 3 years, and OS was 89.4% (95% CI: 78.9-94.8) at 1.5 years and 73.9% (95% CI: 56.6-85.1) at 3 years, as shown in **Supplementary Table 2.** In the > P_50_ group, RFS was 87.0% (95% CI: 76.4-93.0) at 1.5 years and 61.0% (95% CI: 42.6-75.2) at 3 years, and OS was 90.3% (95% CI: 80.7-95.3) at 1.5 years and 74.0% (95% CI: 657.4-84.9) at 3 years. No median survival times for RFS nor OS were reached in either group.

When dividing the CRC patients into two groups according to their relative mtDNA-CN in tumor (< P_50_, *n* = 79; > P_50_, *n* = 80), relapse occurred in 17 patients (21.52%) in the < P_50_ group and in 14 (17.50%) in the > P_50_ group (HR = 0.89, 95% CI: 0.44-1.80; *p* = 0.75), whereas 10 patients (12.66%) died in the < P_50_ group and 16 (20.00%) in the > P_50_ group (HR = 1.64, 95% CI: 0.76-3.54; *p* = 0.21), as shown in **Supplementary Figure 1.** In the < P_50_ group, RFS was 82.1% (95% CI: 70.5-89.5) at 1.5 years and 63.4% (95% CI: 44.3-77.5) at 3 years, and OS was 94.2% (95% CI: 85.3-97.8) at 1.5 years and 76.3% (95% CI: 58.1-87.3) at 3 years, as shown in **Supplementary Table 2.** In the > P_50_ group, RFS was 86.0% (95% CI: 74.7-92.5) at 1.5 years and 73.1% (95% CI: 57.5-83.8) at 3 years, and OS was 85.6% (95% CI: 74.7-92.0) at 1.5 years and 72.0% (95% CI: 56.5-82.8) at 3 years. No median survival times for RFS nor OS were reached in either group.

When dividing the CRC patients into two groups according to their relative mtDNA-CN in ratio tumor/intestinal mucosa (< P_50_, *n* = 79; > P_50_, *n* = 80), relapse occurred in 18 patients (22.78%) in the < P_50_ group and in 13 (16.25%) in the > P_50_ group (HR = 0.78, 95% CI: 0.39-1.59; *p* = 0.50), whereas 14 patients (17.72%) died in the < P_50_ group and 12 (15.00%) in the > P_50_ group (HR = 0.93, 95% CI: 0.43-2.00; *p* = 0.85), as shown in **Supplementary Figure 1.** In the < P_50_ group, RFS was 84.2% (95% CI: 73.3-91.0) at 1.5 years and 63.0% (95% CI: 45.1-76.5) at 3 years, and OS was 90.6% (95% CI: 81.3-95.4) at 1.5 years and 72.5% (95% CI: 56.0-83.7) at 3 years, as shown in **Supplementary Table 2.** In the > P_50_ group, RFS was 83.3% (95% CI: 71.0-90.7) at 1.5 years and 74.9% (95% CI: 59.3-85.3) at 3 years, and OS was 89.0% (95% CI: 78.2-94.7) at 1.5 years and 75.7% (95% CI: 58.4-86.5) at 3 years. No median survival times for RFS nor OS were reached in either group.


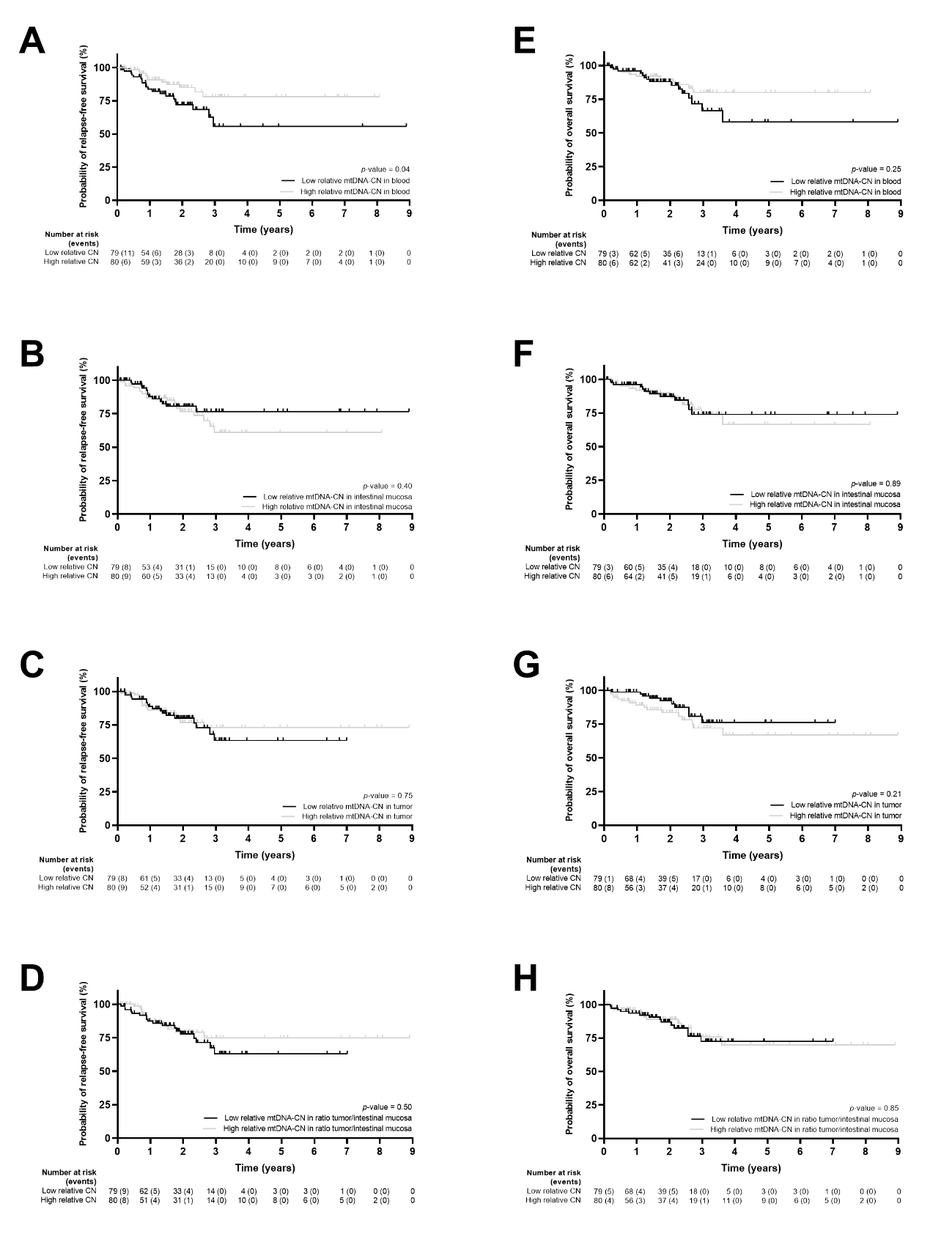


### Supplementary Figure 1. Kaplan-Meier curves for RFS when stratifying CRC patients by relative mtDNA-CN in blood (A), non-tumor intestinal mucosa (B), tumor (C), and ratio tumor/intestinal mucosa (D); and for OS when stratifying by relative mtDNA-CN in blood (E), non-tumor intestinal mucosa (F), tumor (G), and ratio tumor/intestinal mucosa (H).

**Data information:** Patients were stratified into two groups according to the median of the variable. In each graph, the number-at-risk table is shown below the curves, and the *p*-value by the long-rank test is displayed. CN: copy number; CRC: colorectal cancer; mtDNA-CN: mitochondrial DNA copy number.

### **Supplementary Table 3.** Relapse-free and overall survivals of CRC patients according to tissue type when stratified by relative mtDNA-CN.

|  | **Blood** | | **Non-tumor intestinal mucosa** | | **Tumor** | | **Ratio tumor/intestinal mucosa** | |
| --- | --- | --- | --- | --- | --- | --- | --- | --- |
|  | **Low relative mtDNA-CN**  (*n* = 79) | **High relative mtDNA-CN**  (*n* = 80) | **Low relative mtDNA-CN**  (*n* = 79) | **High relative mtDNA-CN**  (*n* = 80) | **Low relative mtDNA-CN**  (*n* = 79) | **High relative mtDNA-CN**  (*n* = 80) | **Low relative mtDNA-CN**  (*n* = 79) | **High relative mtDNA-CN**  (*n* = 80) |
| **Median relapse-free survival (months)** | * | * | * | * | * | * | * | * |
| **Relapse-free survival (%)** | | | | | | | | |
| **At 6 months** | 93.0 (84.0-97.0) | 98.6 (90.8-99.8) | 97.2 (89.3-99.3) | 94.5 (86.0-97.9) | 94.5 (86.0-97.9) | 97.2 (89.4-99.3) | 93.2 (84.3-97.1) | 98.6 (90.2-99.8) |
| **At 12 months** | 83.8 (72.6-90.7) | 91.0 (81.0-95.9) | 87.7 (76.9-93.7) | 87.0 (76.4-93.0) | 88.7 (78.6-94.2) | 86.0 (74.7-92.5) | 87.4 (77.1-93.2) | 87.2 (75.9-93.4) |
| **At 18 months** | 78.6 (66.4-86.8) | 89.2 (78.6-94.7) | 80.5 (68.0-88.5) | 87.0 (76.4-93.0) | 82.1 (70.5-89.5) | 86.0 (74.7-92.5) | 84.2 (73.3-91.0) | 83.3 (71.0-90.7) |
| **At 24 months** | 72.1 (58.6-81.9) | 85.1 (72.9-92.0) | 80.5 (68.0-88.5) | 76.7 (63.4-85.7) | 80.1 (68.0-88.0) | 77.0 (63.1-86.2) | 78.0 (65.2-86.5) | 79.1 (65.9-87.7) |
| **At 30 months** | 68.5 (53.6-79.5) | 81.7 (67.5-90.1) | 76.7 (61.9-86.3) | 73.5 (59.0-83.6) | 72.8 (57.1-83.6) | 77.0 (63.1-86.2) | 71.5 (56.2-82.2) | 79.1 (65.9-87.7) |
| **At 36 months** | 55.8 (34.9-72.4) | 78.1 (62.3-87.9) | 76.7 (61.9-86.3) | 61.0 (42.6-75.2) | 63.4 (44.3-77.5) | 73.1 (57.5-83.8) | 63.0 (45.1-76.5) | 74.9 (59.3-85.3) |
| **At 42 months** | 55.8 (34.9-72.4) | 78.1 (62.3-87.9) | 76.7 (61.9-86.3) | 61.0 (42.6-75.2) | 63.4 (44.3-77.5) | 73.1 (57.5-83.8) | 63.0 (45.1-76.5) | 74.9 (59.3-85.3) |
| **At 48 months** | 55.8 (34.9-72.4) | 78.1 (62.3-87.9) | 76.7 (61.9-86.3) | 61.0 (42.6-75.2) | 63.4 (44.3-77.5) | 73.1 (57.5-83.8) | 63.0 (45.1-76.5) | 74.9 (59.3-85.3) |
| **Median overall survival (months)** | * | * | * | * | * | * | * | * |
| **Overall survival (%)** | | | | | | | | |
| **At 6 months** | 96.0 (88.0-98.7) | 96.2 (88.7-98.8) | 96.1 (88.4-98.7) | 96.1 (88.4-98.7) | 98.7 (91.2-99.8) | 93.5 (85.1-97.3) | 96.2 (88.6-98.7) | 96.1 (88.3-98.7) |
| **At 12 months** | 96.0 (88.0-98.7) | 92.0 (83.1-96.3) | 96.1 (88.4-98.7) | 91.9 (82.8-96.3) | 98.7 (91.2-99.8) | 89.1 (79.2-94.4) | 93.5 (85.2-97.3) | 94.4 (85.6-97.9) |
| **At 18 months** | 88.0 (77.3-93.8) | 92.0 (83.1-96.3) | 89.4 (78.9-94.8) | 90.3 (80.7-95.3) | 94.2 (85.3-97.8) | 85.6 (74.7-92.0) | 90.6 (81.3-95.4) | 89.0 (78.2-94.7) |
| **At 24 months** | 88.0 (77.3-93.8) | 88.3 (77.7-94.0) | 87.4 (76.0-93.5) | 88.5 (78.2-94.1) | 92.4 (82.5-96.8) | 83.6 (72.0-90.7) | 87.1 (76.4-93.1) | 89.0 (78.2-94.7) |
| **At 30 months** | 79.4 (64.6-88.5) | 86.0 (74.3-92.6) | 84.6 (71.9-91.9) | 81.3 (67.9-89.5) | 87.5 (74.8-94.0) | 78.2 (64.4-87.1) | 82.4 (69.9-90.1) | 82.9 (68.4-91.1) |
| **At 36 months** | 66.5 (46.9-80.2) | 80.1 (65.7-89.0) | 73.9 (56.6-85.1) | 74.0 (57.4-84.9) | 76.3 (58.1-87.3) | 72.0 (56.5-82.8) | 72.5 (56.0-83.7) | 75.7 (58.4-86.5) |
| **At 42 months** | 66.5 (46.9-80.2) | 80.1 (65.7-89.0) | 73.9 (56.6-85.1) | 74.0 (57.4-84.9) | 76.3 (58.1-87.3) | 72.0 (56.5-82.8) | 72.5 (56.0-83.7) | 75.7 (58.4-86.5) |
| **At 48 months** | 58.1 (34.6-75.8) | 80.1 (65.7-89.0) | 73.9 (56.6-85.1) | 66.6 (44.8-81.4) | 76.3 (58.1-87.3) | 66.9 (48.8-79.8) | 72.5 (56.0-83.7) | 69.9 (49.6-83.3) |

* Not reported because the median survival time was not reached for this group. Patients were stratified into two groups according to the median of the variable. Results are presented as *value (95% confidence interval)*. CRC: colorectal cancer; mtDNA-CN: mitochondrial DNA copy number.

A total of 159 CRC patients were included in this analysis when employing RTL data, as two patients were excluded due to unclear dates, as shown in **Figure 2.** When dividing the patients into two groups according to their RTL in blood (< P_50_, *n* = 79; > P_50_, *n* = 80), relapse occurred in 13 patients (16.46%) in the < P_50_ group and in 17 (21.25%) in the > P_50_ group (HR = 1.35, 95% CI: 0.66-2.77; *p* = 0.40), whereas 12 patients (15.19%) died in the < P_50_ group and 14 (17.50%) in the > P_50_ group (HR = 1.38, 95% CI: 0.64-2.99; *p* = 0.41), as shown in **Supplementary Figure 2.** In the < P_50_ group, RFS was 81.7% (95% CI: 69.3-89.5) at 1.5 years and 77.5% (95% CI: 64.2-86.4) at 3 years, and OS was 88.3% (95% CI: 78.0-94.0) at 1.5 years and 79.7% (95% CI: 66.4-88.2) at 3 years, as shown in **Supplementary Table 3.** In the > P_50_ group, RFS was 87.5% (95% CI: 77.4-93.3) at 1.5 years and 53.1% (95% CI: 30.5-71.4) at 3 years, and OS was 91.5% (95% CI: 82.0-96.1) at 1.5 years and 64.3% (95% CI: 43.2-79.3) at 3 years. No median survival times for RFS nor OS were reached in either group.

When dividing the CRC patients into two groups according to their RTL in non-tumor intestinal mucosa (< P_50_, *n* = 79; > P_50_, *n* = 80), relapse occurred in 13 patients (16.46%) in the < P_50_ group and in 17 (21.25%) in the > P_50_ group (HR = 1.41, 95% CI: 0.69-2.89; *p* = 0.34), whereas 12 patients (15.19%) died in the < P_50_ group and 14 (17.50%) in the > P_50_ group (HR = 1.32, 95% CI: 0.61-2.86; *p* = 0.47), as shown in **Supplementary Figure 2.** In the < P_50_ group, RFS was 86.7% (95% CI: 76.0-92.9) at 1.5 years and 75.7% (95% CI: 59.1-86.3) at 3 years, and OS was 94.6% (95% CI: 86.2-97.9) at 1.5 years and 73.8% (95% CI: 56.8-84.9) at 3 years, as shown in **Supplementary Table 3.** In the > P_50_ group, RFS was 82.3% (95% CI: 72.2-89.9) at 1.5 years and 61.4% (95% CI: 42.6-75.7) at 3 years, and OS was 85.1% (95% CI: 74.0-91.8) at 1.5 years and 75.3% (95% CI: 59.0-85.8) at 3 years. No median survival times for RFS nor OS were reached in either group.

When dividing the CRC patients into two groups according to their RTL in tumor (< P_50_, *n* = 79; > P_50_, *n* = 80), relapse occurred in 13 patients (16.46%) in the < P_50_ group and in 17 (21.25%) in the > P_50_ group (HR = 1.34, 95% CI: 0.66-2.75; *p* = 0.42), whereas 13 patients (16.46%) died in the < P_50_ group and 13 (16.25%) in the > P_50_ group (HR = 1.00, 95% CI: 0.47-2.17; *p* = 0.99), as shown in **Supplementary Figure 2.** In the < P_50_ group, RFS was 84.3% (95% CI: 72.6-91.3) at 1.5 years and 75.3% (95% CI: 59.3-85.7) at 3 years, and OS was 87.4% (95% CI: 77.1-93.3) at 1.5 years and 77.2% (95% CI: 62.7-86.6) at 3 years, as shown in **Supplementary Table 3.** In the > P_50_ group, RFS was 85.0% (95% CI: 73.8-91.7) at 1.5 years and 61.0% (95% CI: 41.1-76.0) at 3 years, and OS was 92.5% (95% CI: 82.9-96.8) at 1.5 years and 70.5% (95% CI: 51.7-83.1) at 3 years. No median survival times for RFS nor OS were reached in either group.

When dividing the CRC patients into two groups according to their RTL in ratio tumor/intestinal mucosa (< P_50_, *n* = 79; > P_50_, *n* = 80), relapse occurred in 16 patients (20.25%) in the < P_50_ group and in 14 (17.50%) in the > P_50_ group (HR = 0.79, 95% CI: 0.39-1.62; *p* = 0.52), whereas 14 patients (17.72%) died in the < P_50_ group and 12 (15.00%) in the > P_50_ group (HR = 0.77, 95% CI: 0.36-1.66; *p* = 0.50), as shown in **Supplementary Figure 2.** In the < P_50_ group, RFS was 80.6% (95% CI: 68.1-88.6) at 1.5 years and 68.1% (95% CI: 51.2-80.1) at 3 years, and OS was 83.8% (95% CI: 72.6-90.8) at 1.5 years and 75.2% (95% CI: 60.5-85.1) at 3 years, as shown in **Supplementary Table 3.** In the > P_50_ group, RFS was 88.3% (95% CI: 77.9-94.0) at 1.5 years and 70.2% (95% CI: 51.6-82.7) at 3 years, and OS was 95.9% (95% CI: 87.6-98.7) at 1.5 years and 73.3% (95% CI: 54.9-85.1) at 3 years. No median survival times for RFS nor OS were reached in either group.


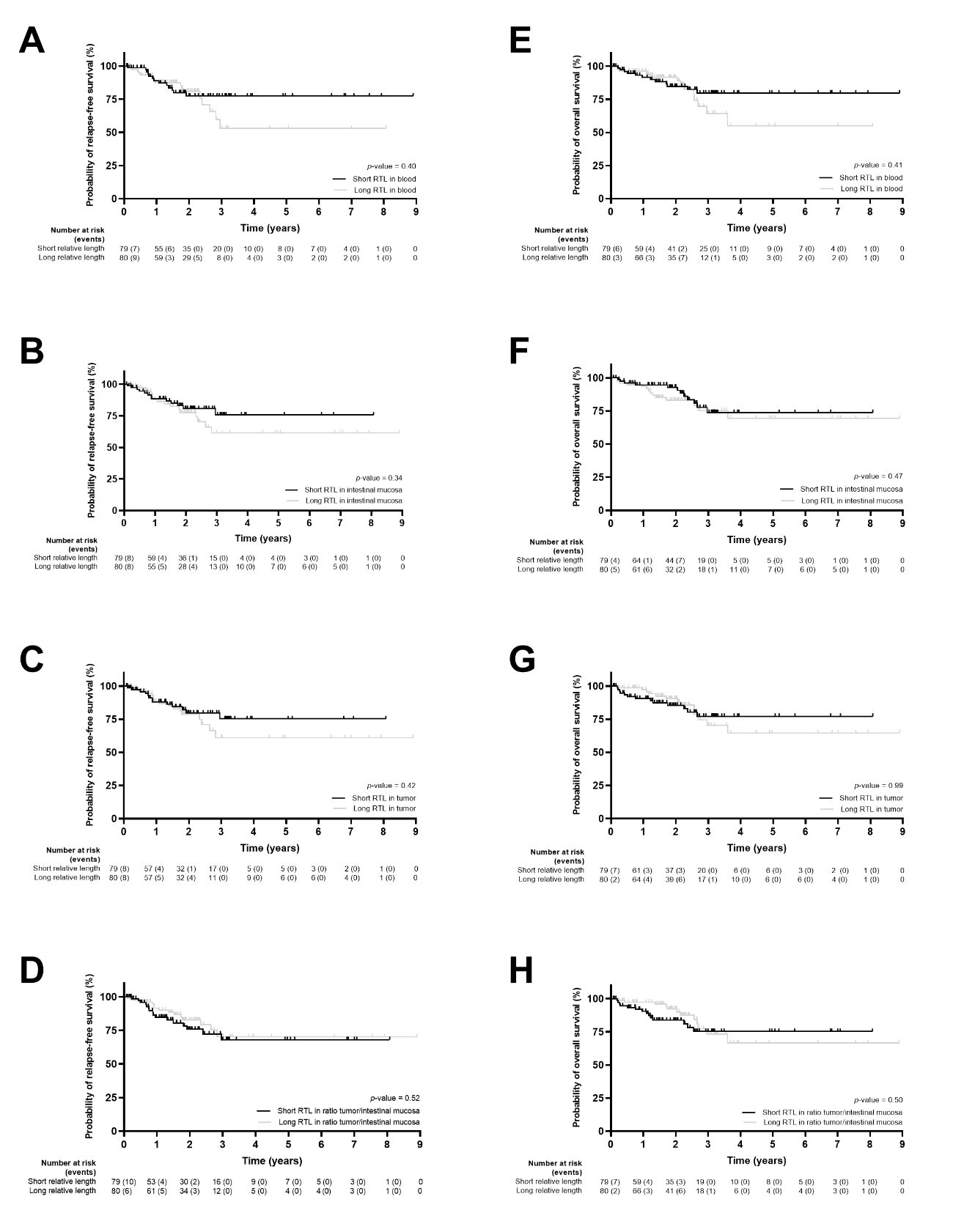


### Supplementary Figure 2. Kaplan-Meier curves for RFS when stratifying CRC patients by RTL in blood (A), non-tumor intestinal mucosa (B), tumor (C), and ratio tumor/intestinal mucosa (D); and for OS when stratifying by RTL in blood (E), non-tumor intestinal mucosa (F), tumor (G), and ratio tumor/intestinal mucosa (H).

**Data information:** Patients were stratified into two groups according to the median of the variable. In each graph, the number-at-risk table is shown below the curves, and the *p*-value by the long-rank test is displayed. CRC: colorectal cancer; RTL: relative telomere length.

### Supplementary Table 4. Relapse-free and overall survivals of CRC patients according to tissue type when stratified by RTL.

|  | **Blood** | | **Non-tumor intestinal mucosa** | | **Tumor** | | **Ratio tumor/intestinal mucosa** | |
| --- | --- | --- | --- | --- | --- | --- | --- | --- |
|  | **Short RTL**  (*n* = 79) | **Long RTL**  (*n* = 80) | **Short RTL**  (*n* = 79) | **Long RTL**  (*n* = 80) | **Short RTL**  (*n* = 79) | **Long RTL**  (*n* = 80) | **Short RTL**  (*n* = 79) | **Long RTL**  (*n* = 80) |
| **Median relapse-free survival (months)** | * | * | * | * | * | * | * | * |
| **Relapse-free survival (%)** | | | | | | | | |
| **At 6 months** | 98.6 (90.7-99.8) | 93.4 (84.8-97.2) | 94.5 (85.9-97.9) | 97.2 (89.4-99.3) | 95.8 (87.5-98.6) | 95.9 (87.9-98.7) | 95.7 (87.3-98.6) | 96.0 (88.1-98.7) |
| **At 12 months** | 88.8 (77.9-94.5) | 87.5 (77.4-93.3) | 88.5 (78.2-94.1) | 87.9 (77.1-93.7) | 88.0 (77.5-93.8) | 88.3 (77.9-94.0) | 84.7 (73.4-91.5) | 91.4 (81.9-96.1) |
| **At 18 months** | 81.7 (69.3-89.5) | 87.5 (77.4-93.3) | 86.7 (76.0-92.9) | 82.3 (70.2-89.9) | 84.3 (72.6-91.3) | 85.0 (73.8-91.7) | 80.6 (68.1-88.6) | 88.3 (77.9-94.0) |
| **At 24 months** | 77.5 (64.2-86.4) | 80.9 (68.3-88.9) | 80.8 (68.3-88.7) | 77.6 (64.1-86.6) | 79.7 (66.6-88.1) | 78.9 (66.1-87.3) | 75.8 (62.0-85.1) | 82.7 (70.7-90.1) |
| **At 30 months** | 77.5 (64.2-86.4) | 70.8 (51.7-83.5) | 80.8 (68.3-88.7) | 70.2 (53.8-81.8) | 79.7 (66.6-88.1) | 71.0 (54.4-82.5) | 72.3 (57.2-82.9) | 79.1 (64.9-88.1) |
| **At 36 months** | 77.5 (64.2-86.4) | 53.1 (30.5-71.4) | 75.7 (59.1-86.3) | 61.4 (42.6-75.7) | 75.3 (59.3-85.7) | 61.0 (41.1-76.0) | 68.1 (51.2-80.1) | 70.2 (51.6-82.7) |
| **At 42 months** | 77.5 (64.2-86.4) | 53.1 (30.5-71.4) | 75.7 (59.1-86.3) | 61.4 (42.6-75.7) | 75.3 (59.3-85.7) | 61.0 (41.1-76.0) | 68.1 (51.2-80.1) | 70.2 (51.6-82.7) |
| **At 48 months** | 77.5 (64.2-86.4) | 53.1 (30.5-71.4) | 75.7 (59.1-86.3) | 61.4 (42.6-75.7) | 75.3 (59.3-85.7) | 61.0 (41.1-76.0) | 68.1 (51.2-80.1) | 70.2 (51.6-82.7) |
| **Median overall survival (months)** | * | * | * | * | * | * | * | * |
| **Overall survival (%)** | | | | | | | | |
| **At 6 months** | 96.0 (88.0-98.7) | 96.2 (88.8-98.8) | 96.0 (88.2-98.7) | 96.2 (88.6-98.8) | 93.4 (84.8-97.2) | 98.8 (91.5-99.8) | 94.7 (86.6-98.0) | 97.4 (90.0-99.3) |
| **At 12 months** | 91.5 (82.0-96.1) | 96.2 (88.8-98.8) | 94.6 (86.2-97.9) | 93.3 (84.7-97.2) | 90.6 (81.3-95.4) | 97.3 (89.4-99.3) | 90.4 (80.9-95.3) | 97.4 (90.0-99.3) |
| **At 18 months** | 88.3 (78.0-94.0) | 91.5 (82.0-96.1) | 94.6 (86.2-97.9) | 85.1 (74.0-91.8) | 87.4 (77.1-93.3) | 92.5 (82.9-96.8) | 83.8 (72.6-90.8) | 95.9 (87.6-98.7) |
| **At 24 months** | 84.8 (73.4-91.5) | 91.5 (82.0-96.1) | 92.8 (83.5-97.0) | 83.0 (71.1-90.3) | 85.5 (74.5-92.0) | 90.6 (80.2-95.7) | 83.8 (72.6-90.8) | 92.3 (82.3-96.8) |
| **At 30 months** | 82.5 (70.0.2-90) | 82.6 (67.6-91.1) | 83.5 (69.9-91.3) | 83.0 (71.1-90.3) | 80.3 (67.0-88.6) | 85.7 (72.9-92.8) | 78.2 (64.5-87.2) | 87.6 (75.2-94.0) |
| **At 36 months** | 79.7 (66.4-88.2) | 64.3 (43.2-79.3) | 73.8 (56.8-84.9) | 75.3 (59.0-85.8) | 77.2 (62.7-86.6) | 70.5 (51.7-83.1) | 75.2 (60.5-85.1) | 73.3 (54.9-85.1) |
| **At 42 months** | 79.7 (66.4-88.2) | 64.3 (43.2-79.3) | 73.8 (56.8-84.9) | 75.3 (59.0-85.8) | 77.2 (62.7-86.6) | 70.5 (51.7-83.1) | 75.2 (60.5-85.1) | 73.3 (54.9-85.1) |
| **At 48 months** | 79.7 (66.4-88.2) | 55.2 (30.3-74.4) | 73.8 (56.8-84.9) | 69.5 (49.9-82.7) | 77.2 (62.7-86.6) | 64.6 (43.6-79.5) | 75.2 (60.5-85.1) | 66.6 (44.8-81.4) |

* Not reported because the median survival time was not reached for this group. Patients were stratified into two groups according to the median of the variable. Results are presented as *value (95% confidence interval)*. CRC: colorectal cancer; RTL: relative telomere length.

## Multivariable analysis

### Supplementary Table 5. Association between relative mtDNA-CN as a continuous variable and risk of recurrence (local recurrence or metachronous metastasis) across tissue types from CRC patients.

|  | **Crude model** | **Model 1^a^** | **Model 2^b^** |
| --- | --- | --- | --- |
|  | **HR (95% CI)** | | |
| **Relative mtDNA-CN in blood** | 0.76 (0.44-1.30) | 0.73 (0.42-1.26) | 0.77 (0.42-1.43) |
| **Relative mtDNA-CN in non-tumor intestinal mucosa** | 1.16 (0.80-1.70) | 1.22 (0.82-1.82) | 1.28 (0.84-1.95) |
| **Relative mtDNA-CN in tumor** | 0.86 (0.61-1.21) | 0.88 (0.62-1.25) | 1.04 (0.70-1.55) |
| **Relative mtDNA-CN in ratio tumor/intestinal mucosa** | 0.29 (0.04-2.31) | 0.27 (0.03-2.29) | 0.34 (0.04-3.02) |

^a^ Adjusted for age and sex. ^b^ Fully adjusted for age, sex, BMI, diabetes, TNM stage, tumor location, tumor histology, MSI, and neoadjuvant chemotherapy received. The TNM staging was classified according to the Union for International Cancer Control (UICC)^5^. Tumor location was classified according to the International Statistical Classification of Diseases and Related Health Problems 10^th^ Revision (ICD-10)^6^, stratifying in right colon (C18.0-18.5), left colon (C18.6-19), and rectum (C20). MSI was classified according to the revised Bethesda Guidelines for hereditary nonpolyposis colorectal cancer (Lynch Syndrome) and Microsatellite Instability^7^, stratifying in stable (0/5 microsatellite sequences of the panel mutated), low instability (1/5 microsatellite sequences mutated), and high instability (≥2/5 microsatellite sequences mutated). BMI: body mass index; CI: confidence interval; CRC: colorectal cancer; HR: hazard ratio; MSI: microsatellite instability; mtDNA-CN: mitochondrial DNA copy number; TNM: Tumor-Node-Metastasis system.

### Supplementary Table 6. Interactions between covariates and relative mtDNA-CN in blood in the risk of recurrence models.

| **Covariate^a^** | **Model** | | |
| --- | --- | --- | --- |
|  | **Crude model** | **Model 1^b^** | **Model 2^c^** |
| **Age** | 0.18 | 0.18 | 0.13 |
| **Sex** | 0.23 | 0.23 | 0.16 |
| **BMI** | 0.64 | 0.64 | 0.53 |
| **Diabetes** | 0.68 | 0.68 | 0.50 |
| **TNM stage** | 0.78 | 0.78 | - |
| **Tumor location** | 0.028 | 0.028 | 0.043 |
| **Tumor histology** | 0.56 | 0.56 | 0.77 |
| **MSI** | 0.45 | 0.45 | 0.50 |
| **Neoadjuvant chemotherapy received** | 0.96 | 0.96 | 0.97 |

^a^ The *p*-values for interaction from the Wald test are shown. ^b^ Adjusted for age and sex. ^c^ Fully adjusted for age, sex, BMI, diabetes, TNM stage, tumor location, tumor histology, MSI, and neoadjuvant chemotherapy received. The TNM staging was classified according to the UICC^5^. Tumor location was classified according to the ICD-10^6^, stratifying in right colon (C18.0-18.5), left colon (C18.6-19), and rectum (C20). MSI was classified according to the revised Bethesda Guidelines for hereditary nonpolyposis colorectal cancer (Lynch Syndrome) and Microsatellite Instability^7^, stratifying in stable (0/5 microsatellite sequences of the panel mutated), low instability (1/5 microsatellite sequences mutated), and high instability (≥2/5 microsatellite sequences mutated). BMI: body mass index; ICD-10: International Statistical Classification of Diseases and Related Health Problems 10^th^ Revision; MSI: microsatellite instability; mtDNA-CN: mitochondrial DNA copy number; TNM: Tumor-Node-Metastasis system; UICC: Union for International Cancer Control.

### Supplementary Table 7. Association between relative mtDNA-CN and risk of recurrence (local recurrence or metachronous metastasis) across tissue types from CRC patients.

|  | ***N*** | **Person-years** | **Number of recurrent cases** | **Incidence rate (per 10 person-years)** | **Crude model** | **Model 1^a^** | **Model 2^b^** |
| --- | --- | --- | --- | --- | --- | --- | --- |
|  |  |  |  |  | **HR (95% CI)** | | |
| **Relative mtDNA-CN in blood** | | | | | | | |
| **Low mtDNA-CN (ref.)** | 79 | 142.04 | 20 | 1.41 | 1.00 | 1.00 | 1.00 |
| **High mtDNA-CN** | 80 | 184.83 | 11 | 0.60 | 0.47 (0.22-0.97)* | 0.43 (0.20-0.91)* | 0.43 (0.20-0.97)* |
| **Relative mtDNA-CN in non-tumor intestinal mucosa** | | | | | | | |
| **Low mtDNA-CN (ref.)** | 79 | 166.41 | 13 | 0.78 | 1.00 | 1.00 | 1.00 |
| **High mtDNA-CN** | 80 | 160.46 | 18 | 1.12 | 1.29 (0.63-2.63) | 1.42 (0.69-2.91) | 1.38 (0.62-3.07) |
| **Relative mtDNA-CN in tumor** | | | | | | | |
| **Low mtDNA-CN (ref.)** | 79 | 159.03 | 17 | 1.07 | 1.00 | 1.00 | 1.00 |
| **High mtDNA-CN** | 80 | 167.84 | 14 | 0.83 | 0.90 (0.44-1.82) | 0.93 (0.46-1.88) | 1.40 (0.60-3.26) |
| **Relative mtDNA-CN in ratio tumor/intestinal mucosa** | | | | | | | |
| **Low mtDNA-CN (ref.)** | 79 | 161.47 | 18 | 1.11 | 1.00 | 1.00 | 1.00 |
| **High mtDNA-CN** | 80 | 165.40 | 13 | 0.79 | 0.82 (0.40-1.68) | 0.80 (0.39-1.64) | 0.90 (0.40-2.05) |

^a^ Adjusted for age and sex. ^b^ Fully adjusted for age, sex, BMI, diabetes, TNM stage, tumor location, tumor histology, MSI, and neoadjuvant chemotherapy received. *, *p* < 0.05. Patients were stratified into two groups according to the median of the variable. The TNM staging was classified according to the UICC^5^. Tumor location was classified according to the ICD-10^6^, stratifying in right colon (C18.0-18.5), left colon (C18.6-19), and rectum (C20). MSI was classified according to the revised Bethesda Guidelines for hereditary nonpolyposis colorectal cancer (Lynch Syndrome) and Microsatellite Instability^7^, stratifying in stable (0/5 microsatellite sequences of the panel mutated), low instability (1/5 microsatellite sequences mutated), and high instability (≥2/5 microsatellite sequences mutated). BMI: body mass index; CI: confidence interval; CRC: colorectal cancer; HR: hazard ratio; ICD-10: International Statistical Classification of Diseases and Related Health Problems 10^th^ Revision; MSI: microsatellite instability; mtDNA-CN: mitochondrial DNA copy number; TNM: Tumor-Node-Metastasis system; UICC: Union for International Cancer Control.

### Supplementary Table 8. Association between relative mtDNA-CN and risk of mortality across tissue types from CRC patients.

|  | ***N*** | **Person-years** | **Number of recurrent cases** | **Incidence rate (per 10 person-years)** | **Crude model** | **Model 1^a^** | **Model 2^b^** |
| --- | --- | --- | --- | --- | --- | --- | --- |
|  |  |  |  |  | **HR (95% CI)** | | |
| **Relative mtDNA-CN in blood** | | | | | | | |
| **Low mtDNA-CN (ref.)** | 79 | 164.00 | 15 | 0.91 | 1.00 | 1.00 | 1.00 |
| **High mtDNA-CN** | 80 | 195.52 | 11 | 0.56 | 0.63 (0.29-1.38) | 0.59 (0.27-1.31) | 0.59 (0.24-1.42) |
| **Relative mtDNA-CN in non-tumor intestinal mucosa** | | | | | | | |
| **Low mtDNA-CN (ref.)** | 79 | 179.28 | 12 | 0.67 | 1.00 | 1.00 | 1.00 |
| **High mtDNA-CN** | 80 | 180.24 | 14 | 0.78 | 1.06 (0.49-2.28) | 1.08 (0.49-2.34) | 1.00 (0.43-2.36) |
| **Relative mtDNA-CN in tumor** | | | | | | | |
| **Low mtDNA-CN (ref.)** | 79 | 176.62 | 10 | 0.57 | 1.00 | 1.00 | 1.00 |
| **High mtDNA-CN** | 80 | 182.90 | 16 | 0.87 | 1.65 (0.74-3.64) | 1.70 (0.77-3.77) | 2.37 (0.98-5.70) |
| **Relative mtDNA-CN in ratio tumor/intestinal mucosa** | | | | | | | |
| **Low mtDNA-CN (ref.)** | 79 | 178.05 | 14 | 0.79 | 1.00 | 1.00 | 1.00 |
| **High mtDNA-CN** | 80 | 181.47 | 12 | 0.66 | 0.93 (0.43-2.01) | 0.94 (0.44-2.05) | 1.35 (0.56-3.23) |

^a^ Adjusted for age and sex. ^b^ Fully adjusted for age, sex, BMI, diabetes, TNM stage, tumor location, tumor histology, MSI, and neoadjuvant chemotherapy received. Patients were stratified into two groups according to the median of the variable. The TNM staging was classified according to the UICC^5^. Tumor location was classified according to the ICD-10^6^, stratifying in right colon (C18.0-18.5), left colon (C18.6-19), and rectum (C20). MSI was classified according to the revised Bethesda Guidelines for hereditary nonpolyposis colorectal cancer (Lynch Syndrome) and Microsatellite Instability^7^, stratifying in stable (0/5 microsatellite sequences of the panel mutated), low instability (1/5 microsatellite sequences mutated), and high instability (≥2/5 microsatellite sequences mutated). BMI: body mass index; CI: confidence interval; CRC: colorectal cancer; HR: hazard ratio; ICD-10: International Statistical Classification of Diseases and Related Health Problems 10^th^ Revision; MSI: microsatellite instability; mtDNA-CN: mitochondrial DNA copy number; TNM: Tumor-Node-Metastasis system; UICC: Union for International Cancer Control.

### Supplementary Table 9. Association between RTL and risk of recurrence (local recurrence or metachronous metastasis) across tissue types from CRC patients.

|  | ***N*** | **Person-years** | **Number of recurrent cases** | **Incidence rate (per 10 person-years)** | **Crude model** | **Model 1^a^** | **Model 2^b^** |
| --- | --- | --- | --- | --- | --- | --- | --- |
|  |  |  |  |  | **HR (95% CI)** | | |
| **RTL in blood** | | | | | | | |
| **Short RTL (ref.)** | 79 | 182.56 | 13 | 0.71 | 1.00 | 1.00 | 1.00 |
| **Long RTL** | 80 | 144.66 | 17 | 1.18 | 1.44 (0.69-2.98) | 1.48 (0.71-3.08) | 1.75 (0.69-4.45) |
| **RTL in non-tumor intestinal mucosa** | | | | | | | |
| **Short RTL (ref.)** | 79 | 162.00 | 13 | 0.80 | 1.00 | 1.00 | 1.00 |
| **Long RTL** | 80 | 165.22 | 17 | 1.03 | 1.44 (0.70-2.97) | 1.42 (0.69-2.95) | 1.54 (0.66-3.62) |
| **RTL in tumor** | | | | | | | |
| **Short RTL (ref.)** | 79 | 160.68 | 13 | 0.81 | 1.00 | 1.00 | 1.00 |
| **Long RTL** | 80 | 166.53 | 17 | 1.02 | 1.37 (0.67-2.84) | 1.30 (0.63-2.69) | 1.27 (0.59-2.74) |
| **RTL in ratio tumor/intestinal mucosa** | | | | | | | |
| **Short RTL (ref.)** | 79 | 161.01 | 16 | 0.99 | 1.00 | 1.00 | 1.00 |
| **Long RTL** | 80 | 166.21 | 14 | 0.84 | 0.81 (0.39-1.66) | 0.82 (0.40-1.69) | 0.83 (0.37-1.85) |

^a^ Adjusted for age and sex. ^b^ Fully adjusted for age, sex, BMI, diabetes, TNM stage, tumor location, tumor histology, MSI, and neoadjuvant chemotherapy received. Patients were stratified into two groups according to the median of the variable. The TNM staging was classified according to the UICC^5^. Tumor location was classified according to the ICD-10^6^, stratifying in right colon (C18.0-18.5), left colon (C18.6-19), and rectum (C20). MSI was classified according to the revised Bethesda Guidelines for hereditary nonpolyposis colorectal cancer (Lynch Syndrome) and Microsatellite Instability^7^, stratifying in stable (0/5 microsatellite sequences of the panel mutated), low instability (1/5 microsatellite sequences mutated), and high instability (≥2/5 microsatellite sequences mutated). BMI: body mass index; CI: confidence interval; CRC: colorectal cancer; HR: hazard ratio; ICD-10: International Statistical Classification of Diseases and Related Health Problems 10^th^ Revision; MSI: microsatellite instability; RTL: relative telomere length; TNM: Tumor-Node-Metastasis system; UICC: Union for International Cancer Control.

### Supplementary Table 10. Association between RTL and risk of mortality across tissue types from CRC patients.

|  | ***N*** | **Person-years** | **Number of recurrent cases** | **Incidence rate (per 10 person-years)** | **Crude model** | **Model 1^a^** | **Model 2^b^** |
| --- | --- | --- | --- | --- | --- | --- | --- |
|  |  |  |  |  | **HR (95% CI)** | | |
| **RTL in blood** | | | | | | | |
| **Short RTL (ref.)** | 79 | 198.02 | 12 | 0.61 | 1.00 | 1.00 | 1.00 |
| **Long RTL** | 80 | 161.64 | 14 | 0.87 | 1.38 (0.64-3.03) | 1.43 (0.65-3.13) | 1.18 (0.44-3.17) |
| **RTL in non-tumor intestinal mucosa** | | | | | | | |
| **Short RTL (ref.)** | 79 | 179.74 | 12 | 0.67 | 1.00 | 1.00 | 1.00 |
| **Long RTL** | 80 | 179.92 | 14 | 0.78 | 1.33 (0.61-2.88) | 1.35 (0.62-2.93) | 1.69 (0.70-4.05) |
| **RTL in tumor** | | | | | | | |
| **Short RTL (ref.)** | 79 | 172.73 | 13 | 0.75 | 1.00 | 1.00 | 1.00 |
| **Long RTL** | 80 | 186.93 | 13 | 0.70 | 1.00 (0.46-2.17) | 1.00 (0.46-2.17) | 0.92 (0.41-2.07) |
| **RTL in ratio tumor/intestinal mucosa** | | | | | | | |
| **Short RTL (ref.)** | 79 | 174.07 | 14 | 0.80 | 1.00 | 1.00 | 1.00 |
| **Long RTL** | 80 | 185.59 | 12 | 0.65 | 0.77 (0.35-1.66) | 0.78 (0.36-1.69) | 0.63 (0.26-1.49) |

^a^ Adjusted for age and sex. ^b^ Fully adjusted for age, sex, BMI, diabetes, TNM stage, tumor location, tumor histology, MSI, and neoadjuvant chemotherapy received. Patients were stratified into two groups according to the median of the variable. The TNM staging was classified according to the UICC^5^. Tumor location was classified according to the ICD-10^6^, stratifying in right colon (C18.0-18.5), left colon (C18.6-19), and rectum (C20). MSI was classified according to the revised Bethesda Guidelines for hereditary nonpolyposis colorectal cancer (Lynch Syndrome) and Microsatellite Instability^7^, stratifying in stable (0/5 microsatellite sequences of the panel mutated), low instability (1/5 microsatellite sequences mutated), and high instability (≥2/5 microsatellite sequences mutated). BMI: body mass index; CI: confidence interval; CRC: colorectal cancer; HR: hazard ratio; ICD-10: International Statistical Classification of Diseases and Related Health Problems 10^th^ Revision; MSI: microsatellite instability; RTL: relative telomere length; TNM: Tumor-Node-Metastasis system; UICC: Union for International Cancer Control.

### Supplementary Table 11. Association between relative mtDNA-CN as a continuous variable and risk of mortality across tissue types from CRC patients.

|  | **Crude model** | **Model 1^a^** | **Model 2^b^** |
| --- | --- | --- | --- |
|  | **HR (95% CI)** | | |
| **Relative mtDNA-CN in blood** | 0.81 (0.45-1.45) | 0.77 (0.43-1.39) | 0.71 (0.35-1.43) |
| **Relative mtDNA-CN in non-tumor intestinal mucosa** | 1.09 (0.73-1.63) | 1.10 (0.73-1.66) | 1.11 (0.69-1.78) |
| **Relative mtDNA-CN in tumor** | 0.92 (0.62-1.38) | 0.95 (0.63-1.42) | 1.06 (0.70-1.59) |
| **Relative mtDNA-CN in ratio tumor/intestinal mucosa** | 0.57 (0.10-3.13) | 0.59 (0.11-3.18) | 0.74 (0.20-2.72) |

^a^ Adjusted for age and sex. ^b^ Fully adjusted for age, sex, BMI, diabetes, TNM stage, tumor location, tumor histology, MSI, and neoadjuvant chemotherapy received. The TNM staging was classified according to the UICC^5^. Tumor location was classified according to the ICD-10^6^, stratifying in right colon (C18.0-18.5), left colon (C18.6-19), and rectum (C20). MSI was classified according to the revised Bethesda Guidelines for hereditary nonpolyposis colorectal cancer (Lynch Syndrome) and Microsatellite Instability^7^, stratifying in stable (0/5 microsatellite sequences of the panel mutated), low instability (1/5 microsatellite sequences mutated), and high instability (≥2/5 microsatellite sequences mutated). BMI: body mass index; CI: confidence interval; CRC: colorectal cancer; HR: hazard ratio; ICD-10: International Statistical Classification of Diseases and Related Health Problems 10^th^ Revision; MSI: microsatellite instability; mtDNA-CN: mitochondrial DNA copy number; TNM: Tumor-Node-Metastasis system; UICC: Union for International Cancer Control.

### Supplementary Table 12. Association between RTL as a continuous variable and risk of recurrence (local recurrence or metachronous metastasis) across tissue types from CRC patients.

|  | **Crude model** | **Model 1^a^** | **Model 2^b^** |
| --- | --- | --- | --- |
|  | **HR (95% CI)** | | |
| **RTL in blood** | 1.15 (0.80-1.65) | 1.17 (0.82-1.69) | 1.22 (0.78-1.91) |
| **RTL in non-tumor intestinal mucosa** | 1.08 (0.75-1.54) | 1.06 (0.73-1.52) | 1.08 (0.70-1.68) |
| **RTL in tumor** | 1.05 (0.79-1.38) | 1.03 (0.79-1.35) | 1.08 (0.79-1.48) |
| **RTL in ratio tumor/intestinal mucosa** | 1.36 (0.04-41.48) | 1.26 (0.06-28.57) | 2.66 (0.06-108.97) |

^a^ Adjusted for age and sex. ^b^ Fully adjusted for age, sex, BMI, diabetes, TNM stage, tumor location, tumor histology, MSI, and neoadjuvant chemotherapy received. The TNM staging was classified according to the UICC^5^. Tumor location was classified according to the ICD-10^6^, stratifying in right colon (C18.0-18.5), left colon (C18.6-19), and rectum (C20). MSI was classified according to the revised Bethesda Guidelines for hereditary nonpolyposis colorectal cancer (Lynch Syndrome) and Microsatellite Instability^7^, stratifying in stable (0/5 microsatellite sequences of the panel mutated), low instability (1/5 microsatellite sequences mutated), and high instability (≥2/5 microsatellite sequences mutated). BMI: body mass index; CI: confidence interval; CRC: colorectal cancer; HR: hazard ratio; ICD-10: International Statistical Classification of Diseases and Related Health Problems 10^th^ Revision; MSI: microsatellite instability; RTL: relative telomere length; TNM: Tumor-Node-Metastasis system; UICC: Union for International Cancer Control.

### Supplementary Table 13. Association between RTL as a continuous variable and risk of mortality across tissue types from CRC patients.

|  | **Crude model** | **Model 1^a^** | **Model 2^b^** |
| --- | --- | --- | --- |
|  | **HR (95% CI)** | | |
| **RTL in blood** | 1.25 (0.87-1.81) | 1.27 (0.88-1.84) | 1.31 (0.82-2.10) |
| **RTL in non-tumor intestinal mucosa** | 1.09 (0.73-1.61) | 1.09 (0.73-1.62) | 1.04 (0.68-1.62) |
| **RTL in tumor** | 0.95 (0.69-1.31) | 0.94 (0.68-1.30) | 0.94 (0.68-1.31) |
| **RTL in ratio tumor/intestinal mucosa** | 0.27 (0.01-12.25) | 0.25 (0.01-10.44) | 0.30 (0.00-19.87) |

^a^ Adjusted for age and sex. ^b^ Fully adjusted for age, sex, BMI, diabetes, TNM stage, tumor location, tumor histology, MSI, and neoadjuvant chemotherapy received. The TNM staging was classified according to the UICC^5^. Tumor location was classified according to the ICD-10^6^, stratifying in right colon (C18.0-18.5), left colon (C18.6-19), and rectum (C20). MSI was classified according to the revised Bethesda Guidelines for hereditary nonpolyposis colorectal cancer (Lynch Syndrome) and Microsatellite Instability^7^, stratifying in stable (0/5 microsatellite sequences of the panel mutated), low instability (1/5 microsatellite sequences mutated), and high instability (≥2/5 microsatellite sequences mutated). BMI: body mass index; CI: confidence interval; CRC: colorectal cancer; HR: hazard ratio; ICD-10: International Statistical Classification of Diseases and Related Health Problems 10^th^ Revision; MSI: microsatellite instability; RTL: relative telomere length; TNM: Tumor-Node-Metastasis system; UICC: Union for International Cancer Control.

## Measurement of relative mtDNA copy number

When including the age of the colorectal cancer (CRC) patients in the analysis, there was a non-significant positive correlation between relative mitochondrial DNA copy number (mtDNA-CN) in blood and age (range: 39-87 years old, *n* = 159; ρ = 0.08, *p* = 0.30). However, when stratifying the patients into two groups, the correlation turned to be negative when age < 55 years (range: 39-54 years old, *n* = 22; ρ = -0.05, *p* = 0.81), and significantly positive when age > 55 years (range: 56-87 years old, *n* = 137; **ρ = 0.17, *p* = 0.046**), as shown in **Supplementary** **Figure 3.** A weak but significant positive correlation was found between relative mtDNA-CN in non-tumor intestinal mucosa and age (**ρ = 0.17, *p* = 0.03**), but the significance was only maintained when age > 55 years (**ρ = 0.17, *p* = 0.04**) and not when age < 55 years (ρ = -0.07, *p* = 0.77). A non-significant positive correlation was found between relative mtDNA-CN in tumor and age (ρ = 0.13, *p* = 0.10), even when dividing in age < 55 years (ρ = 0.13, *p* = 0.57), and age > 55 years (ρ = 0.10, *p* = 0.27). Lastly, a non-significant negative correlation was found between relative mtDNA-CN in ratio tumor/intestinal mucosa and age (ρ = -0.04, *p* = 0.62). This fact was maintained when age > 55 years (ρ = -0.08, *p* = 0.33), but the correlation turned to be positive when dividing in age < 55 years (ρ = 0.02, *p* = 0.94).


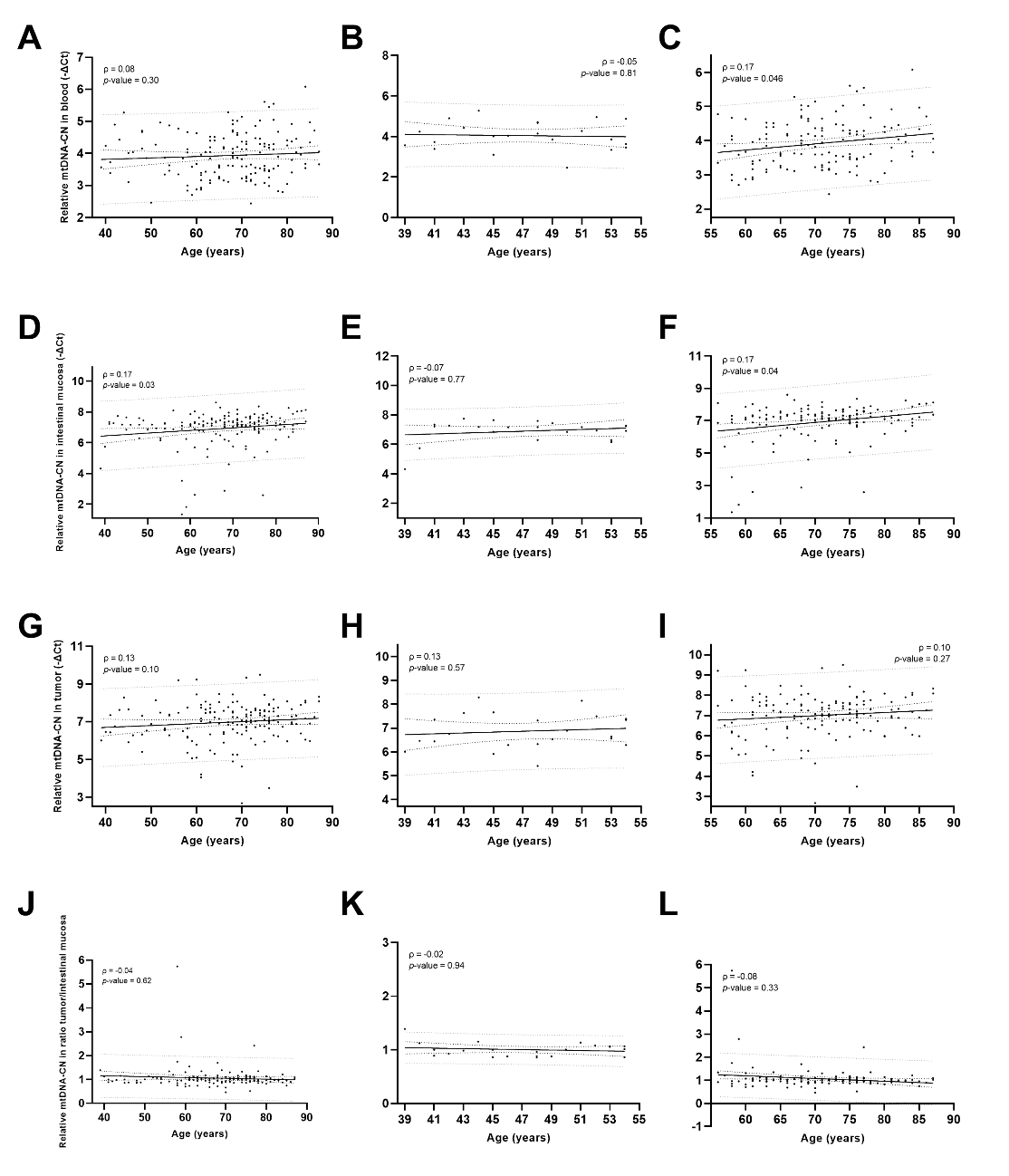


### Supplementary Figure 3. Correlation between relative mtDNA-CN and age in CRC patients across tissue types. Patients were stratified into two groups: age < 55 years (range: 39-54 years old, *n* = 22), and age > 55 years (range: 56-87 years old, *n* = 137) (total range: 39-87 years old, total sample size: *n* = 159). The figure is divided in panels as follows: correlation between age and relative mtDNA-CN in blood (A), age < 55 years and relative mtDNA-CN in blood (B), age > 55 years and relative mtDNA-CN in blood (C), age and relative mtDNA-CN in non-tumor intestinal mucosa (D), age < 55 years and relative mtDNA-CN in non-tumor intestinal mucosa (E), age > 55 years and relative mtDNA-CN in non-tumor intestinal mucosa (F), age and relative mtDNA-CN in tumor (G), age < 55 years and relative mtDNA-CN in tumor (H), age > 55 years and relative mtDNA-CN in tumor (I), age and relative mtDNA-CN in ratio tumor/intestinal mucosa (J), age < 55 years and relative mtDNA-CN in ratio tumor/intestinal mucosa (K), and age > 55 years and relative mtDNA-CN in ratio tumor/intestinal mucosa (L).

**Data information:** In each graph, both Spearman’s rho (ρ) correlation coefficient and the *p*-value are shown, together with the 95% confidence intervals (dark dotted lines) and the 95% prediction intervals (gray dotted lines). CRC: colorectal cancer; Ct: cycle threshold; mtDNA-CN: mitochondrial DNA copy number.

The relative mtDNA-CN of the individual tissues and the ratio tumor/intestinal mucosa from CRC patients were also compared according to the baseline sociodemographic and clinicopathological characteristics of patients, as shown in **Supplementary Figure 4.** Like that, when stratifying patients by sex, the relative mtDNA-CN was not significantly different neither in blood (male: *n* = 107, $\bar{x}$ = 4.00, s = 0.71; female: *n* = 54, $\bar{x}$ = 3.84, s = 0.63; *p* = 0.19), non-tumor intestinal mucosa (male: P_50_ = 7.18, IQR = 6.85-7.47; female: P_50_ = 7.18, IQR = 6.76-7.40; *p* = 0.86) nor tumor (male: P_50_ = 7.09, IQR = 6.42-7.55; female: P_50_ = 7.13, IQR = 6.50-7.66; *p* = 0.55). A non-significant negative correlation was found between body mass index (BMI) and relative mtDNA-CN in blood (*n* = 160; ρ = -0.13, *p* = 0.10), whereas non-significant positive correlations were found between BMI and relative mtDNA-CN in non-tumor intestinal mucosa (*n* = 160; ρ = 0.02, *p* = 0.78), tumor (*n* = 160; ρ = 0.05, *p* = 0.50), and ratio tumor/intestinal mucosa (*n* = 160; ρ = 0.03, *p* = 0.68). When stratifying patients by the presence of diabetes, the relative mtDNA-CN was not significantly different neither in blood (diabetes: *n* = 37, $\bar{x}$ = 3.89, s = 0.70; no diabetes: *n* = 124, $\bar{x}$ = 3.96, s = 0.69; *p* = 0.59), non-tumor intestinal mucosa (diabetes: P_50_ = 7.21, IQR = 6.97-7.56; no diabetes: P_50_ = 7.17, IQR = 6.85-7.43; *p* = 0.35) nor tumor (diabetes: P_50_ = 7.17, IQR = 6.79-7.65; no diabetes: P_50_ = 7.07, IQR = 6.33-7.55; *p* = 0.22). When stratifying patients by tumor-node-metastasis (TNM) staging, the relative mtDNA-CN was not significantly different neither in blood (TNM I+II: *n* = 87, $\bar{x}$ = 3.97, s = 0.71; TNM III+IV: *n* = 72, $\bar{x}$ = 3.92, s = 0.66; *p* = 0.64), non-tumor intestinal mucosa (TNM I+II: P_50_ = 7.17, IQR = 6.77-7.39; TNM III+IV: P_50_ = 7.24, IQR = 6.90-7.57; *p* = 0.13) nor tumor (TNM I+II: P_50_ = 7.17, IQR = 6.46-7.62; TNM III+IV: P_50_ = 7.04, IQR = 6.38-7.61; *p* = 0.72). When stratifying patients by the presence of distant metastasis, the relative mtDNA-CN was not significantly different neither in blood (metastasis: *n* = 26, $\bar{x}$ = 4.03, s = 0.66; no metastasis: *n* = 133, $\bar{x}$ = 3.94, s = 0.69; *p* = 0.51), non-tumor intestinal mucosa (metastasis: P_50_ = 7.30, IQR = 6.90-7.61; no metastasis: P_50_ = 7.18, IQR = 6.85-7.45; *p* = 0.49) nor tumor (metastasis: P_50_ = 7.14, IQR = 6.42-7.60; no metastasis: P_50_ = 7.08, IQR = 6.45-7.62; *p* = 0.97).

When stratifying CRC patients by tumor location, the relative mtDNA-CN was not significantly different neither in blood (right colon: *n* = 39, $\bar{x}$ = 4.07, s = 0.78; left colon: *n* = 70, $\bar{x}$ = 3.91, s = 0.67; rectum: *n* = 52, $\bar{x}$ = 3.90, s = 0.64; *p* = 0.45), non-tumor intestinal mucosa (right colon: P_50_ = 7.12, IQR = 6.87-7.35; left colon: P_50_ = 7.23, IQR = 6.70-7.60; rectum: P_50_ = 7.17, IQR = 6.95-7.44; *p* = 0.62) nor tumor (right colon: P_50_ = 7.04, IQR = 6.05-7.55; left colon: P_50_ = 7.27, IQR = 6.73-7.69; rectum: P_50_ = 6.92, IQR = 6.38-7.50; *p* = 0.16). When stratifying patients by tumor histology, the relative mtDNA-CN was not significantly different neither in blood (mucinous: *n* = 18, $\bar{x}$ = 3.99, s = 0.67; mixed: *n* = 13, $\bar{x}$ = 3.97, s = 0.90; other: *n* = 128, $\bar{x}$ = 3.93, s = 0.66; *p* = 0.92), non-tumor intestinal mucosa (mucinous: P_50_ = 6.87, IQR = 5.39-7.34; mixed: P_50_ = 7.05, IQR = 6.75-7.25; other: P_50_ = 7.22, IQR = 6.91-7.55; *p* = 0.06) nor tumor (mucinous: P_50_ = 6.95, IQR = 6.22-7.54; mixed: P_50_ = 7.18, IQR = 6.68-7.99; other: P_50_ = 7.11, IQR = 6.52-7.62; *p* = 0.52). When stratifying patients by tumor microsatellite instability (MSI), the relative mtDNA-CN was not significantly different neither in blood (stable: *n* = 90, P_50_ = 3.85, IQR = 3.39-4.38; low instability: *n* = 5, P_50_ = 3.96, IQR = 3.42-4.17; high instability: *n* = 5, P_50_ = 3.17, IQR = 3.15-3.92; *p* = 0.33), non-tumor intestinal mucosa (stable: P_50_ = 7.19, IQR = 6.81-7.45; low instability: P_50_ = 7.03, IQR = 6.83-7.22; high instability: P_50_ = 7.38, IQR = 4.89-7.69; *p* = 0.59) nor tumor (stable: P_50_ = 7.10, IQR = 6.53-7.59; low instability: P_50_ = 7.35, IQR = 6.19-7.85; high instability: P_50_ = 6.79, IQR = 4.76-8.48; *p* = 0.87). Lastly, when stratifying patients by whether neoadjuvant chemotherapy was received, the relative mtDNA-CN was not significantly different neither in blood (received: *n* = 42, $\bar{x}$ = 3.87, s = 0.62; not received: *n* = 119, $\bar{x}$ = 3.97, s = 0.71; *p* = 0.33), non-tumor intestinal mucosa (received: P_50_ = 7.17, IQR = 6.91-7.40; not received: P_50_ = 7.18, IQR = 6.77-7.50; *p* = 0.93) nor tumor (received: P_50_ = 6.90, IQR = 6.27-7.49; not received: P_50_ = 7.18, IQR = 6.53-7.65; *p* = 0.10).


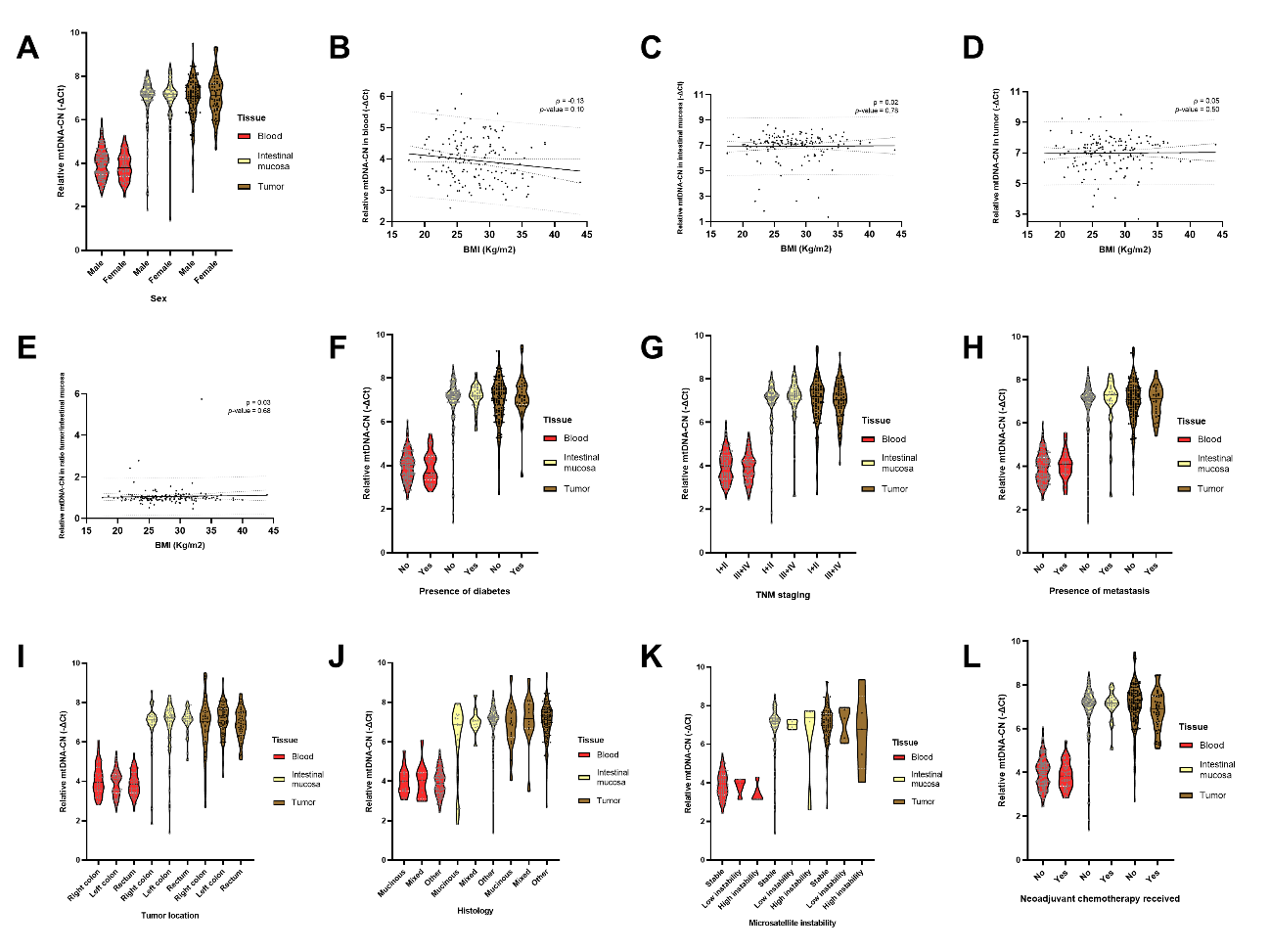


### Supplementary Figure 4. Relative mtDNA-CN according to tissue type when stratifying CRC patients by sociodemographic variables and clinicopathological data: sex (A), presence of diabetes (F), TNM staging (G), presence of distant metastasis (H), tumor location (I), histology (J), microsatellite instability (K), and neoadjuvant chemotherapy received (L). The correlations between BMI and relative mtDNA-CN in blood (B), non-tumor intestinal mucosa (C), tumor (D), and ratio tumor/intestinal mucosa (E) are also shown. The TNM staging was classified according to the UICC^5^. Tumor location was classified according to the ICD-10^6^, stratifying in right colon (C18.0-18.5), left colon (C18.6-19), and rectum (C20). MSI was classified according to the revised Bethesda Guidelines for hereditary nonpolyposis colorectal cancer (Lynch Syndrome) and Microsatellite Instability^7^, stratifying in stable (0/5 microsatellite sequences of the panel mutated), low instability (1/5 microsatellite sequences mutated), and high instability (≥2/5 microsatellite sequences mutated).

**Data information:** Sample sizes of the stratified groups: *n*_male_ = 107, *n*_female_ = 54; *n*_BMI_ = 160; *n*_diabetes_ = 37, *n*_no diabetes_ = 124; *n*_TNM I+II_ = 87, *n*_TNM III+IV_ = 72; *n*_metastasis_ = 26, *n*_no metastasis_ = 133; *n*_right colon_ = 39, *n*_left colon_ = 70, *n*_rectum_ = 52; *n*_mucinous_ = 18, *n*_mixed_ = 13, *n*_other_ = 128; *n*_stable_ = 90, *n*_low instability_ = 5, and *n*_high instability_ = 5; *n*_received_ = 42, *n*_not received_ = 119. Statistical differences were assessed with the unpaired Student *t-*test or the Mann-Whitney *U* test in panels **A**, **F**, **G**, **H**, and **L**; and with ordinary one-way ANOVA or the Kruskal-Wallis test both followed by pairwise multiple comparisons correcting the resultant *p*-values with the method of Benjamini and Hochberg controlling the FDR at 5% in panels **I**, **J**, and **K**. Both Spearman’s rho (ρ) correlation coefficient and the *p*-value are shown, together with the 95% confidence intervals (dark dotted lines) and the 95% prediction intervals (gray dotted lines), in panels **B**, **C**, **D**, and **E**. ANOVA: analysis of variance; BMI: body mass index; CRC: colorectal cancer; Ct: cycle threshold; FDR: false discovery rate; ICD-10: International Statistical Classification of Diseases and Related Health Problems 10^th^ Revision; mtDNA-CN: mitochondrial DNA copy number; TNM: Tumor-Node-Metastasis system; UICC: Union for International Cancer Control.

## Measurement of relative telomere length

When including the age of the CRC patients in the analysis, there was a non-significant positive correlation between relative telomere length (RTL) in blood and age (range: 39-87 years old, *n* = 159; ρ = 0.01, *p* = 0.92). When dividing the patients into two groups, this fact was maintained when age > 55 years (range: 56-87 years old, *n* = 137; ρ = 0.05, *p* = 0.54), but the correlation turned to be negative when age < 55 years (range: 39-54 years old, *n* = 22; ρ = -0.37, *p* = 0.09), as shown in **Supplementary Figure 5**. The correlation between RTL in non-tumor intestinal mucosa and age was also non-significant (ρ = -0.15, *p* = 0.06), even when dividing in age < 55 years (ρ = -0.33, *p* = 0.13), but turned to be significant when age > 55 years (**ρ = -0.23, *p* = 0.007**). A significant negative correlation was found between RTL in tumor and age (**ρ = -0.16, *p* = 0.046**), which was maintained when age >55 years (**ρ = -0.18, *p* = 0.04**) but turned to be non-significant when age < 55 years (ρ = -0.08, *p* = 0.72). Lastly, a non-significant negative correlation was found between RTL in ratio tumor/intestinal mucosa and age (ρ = -0.03, *p* = 0.71), even when age >55 years (ρ = -0.004, *p* = 0.96), but turned to be positive when age < 55 years (ρ = 0.02, *p* = 0.93).


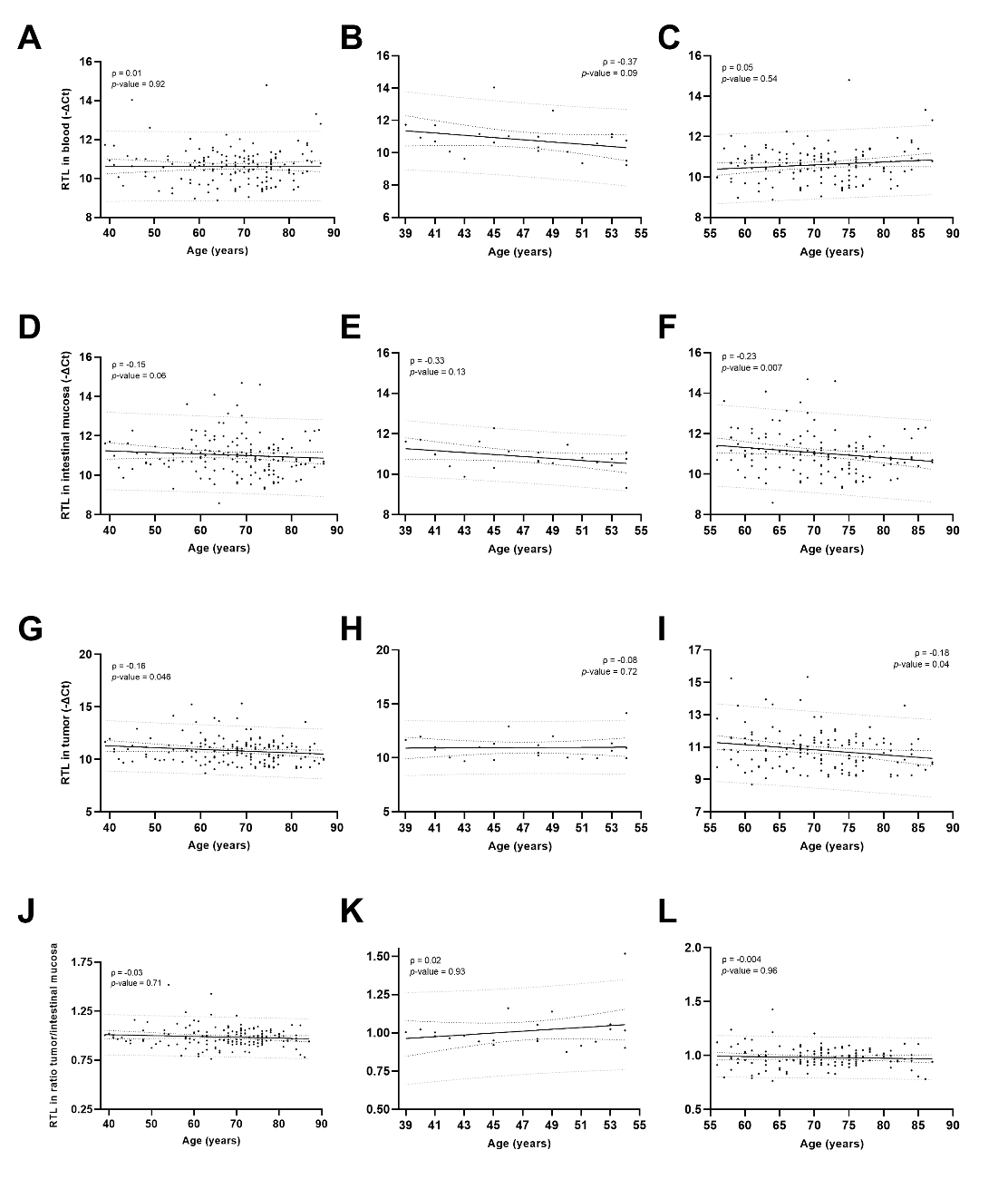


### Supplementary Figure 5. Correlation between RTL and age in CRC patients across tissue types. Patients were stratified into two groups: age < 55 years (range: 39-54 years old, *n* = 22), and age > 55 years (range: 56-87 years old, *n* = 137) (total range: 39-87 years old, total sample size: *n* = 159). The figure is divided in panels as follows: correlation between age and RTL in blood (A), age < 55 years and RTL in blood (B), age > 55 years and RTL in blood (C), age and RTL in non-tumor intestinal mucosa (D), age < 55 years and RTL in non-tumor intestinal mucosa (E), age > 55 years and RTL in non-tumor intestinal mucosa (F), age and RTL in tumor (G), age < 55 years and RTL in tumor (H), age > 55 years and RTL in tumor (I), age and RTL in ratio tumor/intestinal mucosa (J), age < 55 years and RTL in ratio tumor/intestinal mucosa (K), and age > 55 years and RTL in ratio tumor/intestinal mucosa (L).

**Data information:** In each graph, both Spearman’s rho (ρ) correlation coefficient and the *p*-value are shown, together with the 95% confidence intervals (dark dotted lines) and the 95% prediction intervals (gray dotted lines). CRC: colorectal cancer; Ct: cycle threshold; RTL: relative telomere length.

The RTL of the individual tissues and the ratio tumor/intestinal mucosa from CRC patients were also compared according to the baseline sociodemographic and clinicopathological characteristics of patients, as shown in **Supplementary Figure 6.** Like that, when stratifying patients by sex, the RTL was not significantly different neither in blood (male: *n* = 108, P_50_ = 10.62, IQR = 9.92-11.02; female: *n* = 53, P_50_ = 10.82, IQR = 10.29-11.16; *p* = 0.12), non-tumor intestinal mucosa (male: P_50_ = 10.87, IQR = 10.39-11.48; female: P_50_ = 10.99, IQR = 10.56-11.40; *p* = 0.54) nor tumor (male: P_50_ = 10.68, IQR = 9.96-11.43; female: P_50_ = 10.88, IQR = 9.80-11.45; *p* = 0.98). A non-significant negative correlation was found between BMI and RTL in blood (*n* = 160; ρ = -0.03, *p* = 0.71), whereas a non-significant positive correlation was found between BMI and RTL in non-tumor intestinal mucosa (ρ = 0.02, *p* = 0.80), tumor (ρ = 0.10, *p* = 0.22), and ratio tumor/intestinal mucosa (ρ = 0.11, *p* = 0.17). When stratifying patients by the presence of diabetes, the RTL was not significantly different neither in blood (diabetes: *n* = 36, P_50_ = 10.42, IQR = 9.81-10.88; no diabetes: *n* = 125, P_50_ = 10.71, IQR = 10.09-11.11; *p* = 0.10), non-tumor intestinal mucosa (diabetes: P_50_ = 10.76, IQR = 10.15-11.46; no diabetes: P_50_ = 10.96, IQR = 10.55-11.46; *p* = 0.22) nor tumor (diabetes: P_50_ = 10.92, IQR = 9.78-11.68; no diabetes: P_50_ = 10.77, IQR = 9.98-11.38; *p* = 0.94). When stratifying patients by TNM staging, the RTL in blood was significantly longer in TNM III+IV than in TNM I+II (TNM I+II: *n* = 87, P_50_ = 10.58, IQR = 9.90-10.94; TNM III+IV: *n* = 72, P_50_ = 10.82, IQR = 10.11-11.26; ***p* = 0.01**), but not significantly different in non-tumor intestinal mucosa (TNM I+II: P_50_ = 10.94, IQR = 10.40-11.41; TNM III+IV: P_50_ = 10.96, IQR = 10.47-11.59; *p* = 0.61) nor tumor (TNM I+II: P_50_ = 10.77, IQR = 9.96-11.50; TNM III+IV: P_50_ = 10.84, IQR = 9.91-11.42; *p* = 0.89). When stratifying patients by the presence of distant metastasis, the RTL in blood was significantly longer in patients with metastasis (metastasis: *n* = 26, P_50_ = 11.92, IQR = 10.33-11.47; no metastasis: *n* = 133, P_50_ = 10.63, IQR = 9.92-11.02; ***p* = 0.03**), but significantly different neither in non-tumor intestinal mucosa (metastasis: P_50_ = 11.00, IQR = 10.63-11.41; no metastasis: P_50_ = 10.94, IQR = 10.40-11.51; *p* = 0.89) nor tumor (metastasis: P_50_ = 10.80, IQR = 9.80-11.58; no metastasis: P_50_ = 10.83, IQR = 9.97-11.43; *p* = 0.92).

When stratifying CRC patients by tumor location, the RTL was shown to be dependent on location in non-tumor intestinal mucosa (***p* < 0.01**). Like that, relative levels were significantly longer in right colon than in rectum (right colon: *n* = 39, P_50_ = 11.17, IQR = 10.80-11.98; rectum: *n* = 51, P_50_ = 10.68, IQR = 10.19-11.17; ***p* < 0.01**), but not significantly different between right colon and left colon (left colon: *n* = 71, P_50_ = 10.96, IQR = 10.43-11.70; *p* = 0.07) nor left colon and rectum (*p* = 0.06). The RTL was not significantly different neither in blood (right colon: P_50_ = 10.73, IQR = 10.39-11.09; left colon: P_50_ = 10.69, IQR = 10.01-11.00; rectum: P_50_ = 10.63, IQR = 9.90-11.15; *p* = 0.71) nor tumor (right colon: P_50_ = 10.58, IQR = 9.87-11.09; left colon: P_50_ = 11.16, IQR = 9.96-11.53; rectum: P_50_ = 10.67, IQR = 9.77-11.44; *p* = 0.24). When stratifying patients by tumor histology, the RTL was not significantly different neither in blood (mucinous: *n* = 17, P_50_ = 10.76, IQR = 10.39-10.95; mixed: *n* = 13, P_50_ = 10.71, IQR = 10.00-10.88; other: *n* = 129, P_50_ = 10.66, IQR = 9.93-11.14; *p* = 0.97), non-tumor intestinal mucosa (mucinous: P_50_ = 11.17, IQR = 10.46-11.66; mixed: P_50_ = 10.74, IQR = 9.93-11.37; other: P_50_ = 10.94, IQR = 10.43-11.43; *p* = 0.60) nor tumor (mucinous: P_50_ = 11.25, IQR = 10.36-11.54; mixed: P_50_ = 0.83, IQR = 9.38-11.35; other: P_50_ = 10.83, IQR = 9.96-11.41; *p* = 0.17). When stratifying patients by tumor MSI, the RTL was not significantly different neither in blood (stable: *n* = 90, P_50_ = 10.84, IQR = 10.57-11.28; low instability: *n* = 5, P_50_ = 10.90, IQR = 10.40-11.17; high instability: *n* = 5, P_50_ = 10.43, IQR = 9.64-11.26; *p* = 0.57), non-tumor intestinal mucosa (stable: P_50_ = 11.03, IQR = 10.69-11.78; low instability: P_50_ = 10.80, IQR = 10.41-12.59; high instability: P_50_ = 10.83, IQR = 9.77-11.77; *p* = 0.67) nor tumor (stable: P_50_ = 10.93, IQR = 10.02-11.54; low instability: P_50_ = 10.85, IQR = 8.99-12.61; high instability: P_50_ = 10.84, IQR = 9.78-11.65; *p* = 0.84). Lastly, when stratifying patients by whether neoadjuvant chemotherapy was received, the RTL was not significantly different neither in blood (received: *n* = 41, P_50_ = 10.76, IQR = 9.96-11.15; not received: *n* = 120, P_50_ = 10.64, IQR = 10.02-11.08; *p* = 0.71), non-tumor intestinal mucosa (received: P_50_ = 10.75, IQR = 10.22-11.19; not received: P_50_ = 10.99, IQR = 10.51-11.59; *p* = 0.08) nor tumor (received: P_50_ = 10.84, IQR = 10.01-11.58; not received: P_50_ = 10.73, IQR = 9.89-11.41; *p* = 0.50).


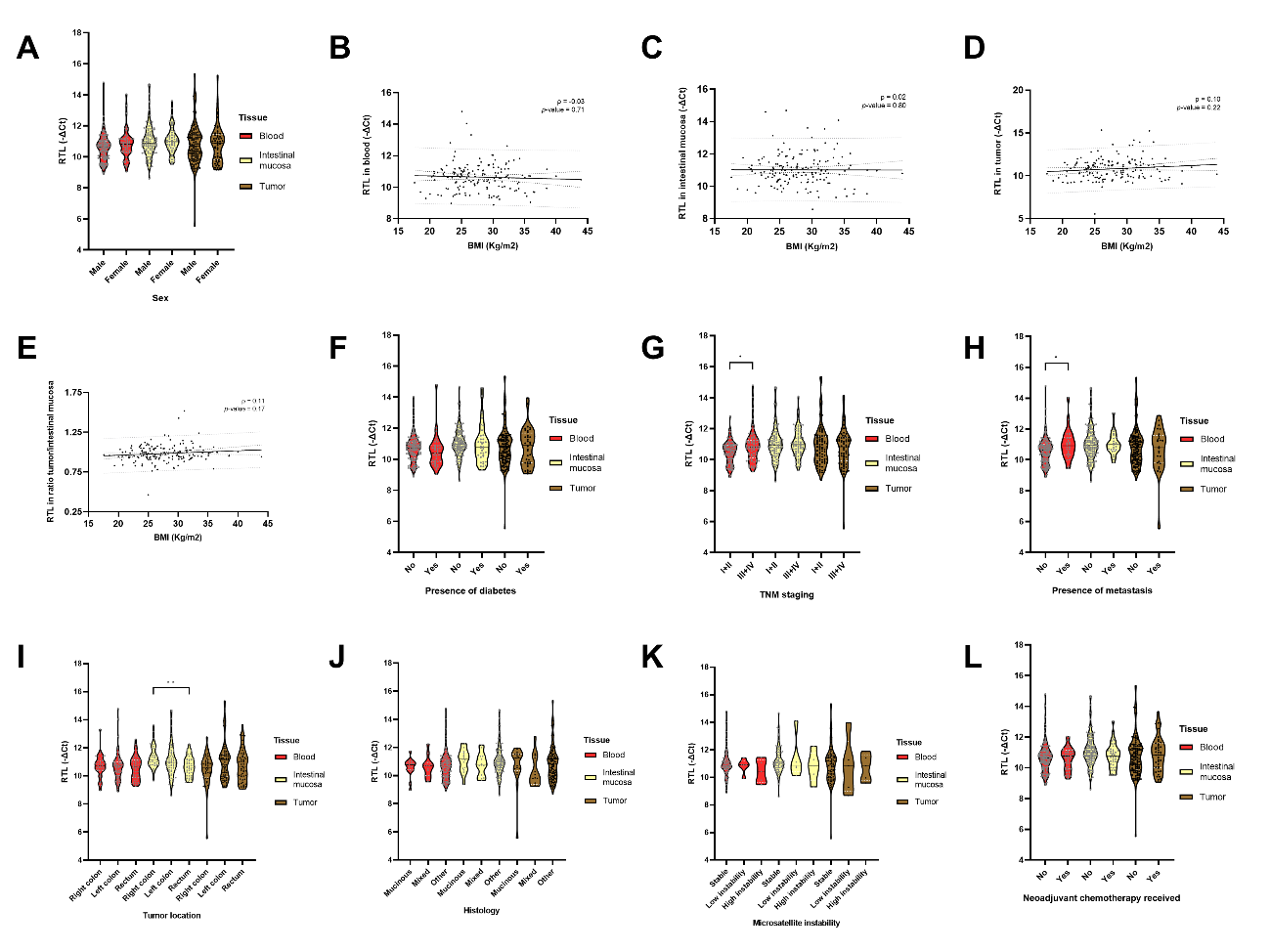


### Supplementary Figure 6. RTL according to tissue type when stratifying CRC patients by sociodemographic variables and clinicopathological data: sex (A), presence of diabetes (F), TNM staging (G), presence of distant metastasis (H), tumor location (I), histology (J), microsatellite instability (K), and neoadjuvant chemotherapy received (L). The correlations between BMI and RTL in blood (B), non-tumor intestinal mucosa (C), tumor (D), and ratio tumor/intestinal mucosa (E) are also shown. The TNM staging was classified according to the UICC^5^. Tumor location was classified according to the ICD-10^6^, stratifying in right colon (C18.0-18.5), left colon (C18.6-19), and rectum (C20). MSI was classified according to the revised Bethesda Guidelines for hereditary nonpolyposis colorectal cancer (Lynch Syndrome) and Microsatellite Instability^7^, stratifying in stable (0/5 microsatellite sequences of the panel mutated), low instability (1/5 microsatellite sequences mutated), and high instability (≥2/5 microsatellite sequences mutated).

**Data information:** Sample sizes of the stratified groups: *n*_male_ = 108, *n*_female_ = 53; *n*_BMI_ = 160; *n*_diabetes_ = 36, *n*_no diabetes_ = 125; *n*_TNM I+II_ = 87, *n*_TNM III+IV_ = 72; *n*_metastasis_ = 26, *n*_no metastasis_ = 133; *n*_right colon_ = 39, *n*_left colon_ = 71, *n*_rectum_ = 51; *n*_mucinous_ = 17, *n*_mixed_ = 13, *n*_other_ = 129; *n*_stable_ = 90, *n*_low instability_ = 5, and *n*_high instability_ = 5; *n*_received_ = 41, *n*_not received_ = 120. Statistical differences were assessed with the unpaired Student *t-*test or the Mann-Whitney *U* test in panels **A**, **F**, **G**, **H**, and **L**; and with ordinary one-way ANOVA or the Kruskal-Wallis test both followed by pairwise multiple comparisons correcting the resultant *p*-values with the method of Benjamini and Hochberg controlling the FDR at 5% in panels **I**, **J**, and **K**. Both Spearman’s rho (ρ) correlation coefficient and the *p*-value are shown, together with the 95% confidence intervals (dark dotted lines) and the 95% prediction intervals (gray dotted lines), in panels **B**, **C**, **D**, and **E**. *, *p* < 0.05; **, *p* < 0.01. ANOVA: analysis of variance; BMI: body mass index; CRC: colorectal cancer; Ct: cycle threshold; FDR: false discovery rate; ICD-10: International Statistical Classification of Diseases and Related Health Problems 10^th^ Revision; RTL: relative telomere length; TNM: Tumor-Node-Metastasis system; UICC: Union for International Cancer Control.

# List of abbreviations

***ALB:*** albumin gene

**ANOVA:** analysis of variance

**BMI:** body mass index

**CI:** confidence interval

**CN:** copy number

**CRC:** colorectal cancer

**Ct:** cycle threshold

**FDR:** false discovery rate

**HR:** hazard ratio

**ICD-10:** International Statistical Classification of Diseases and Related Health Problems 10^th^ Revision

**IQR:** interquartile range

**MSI:** microsatellite instability

**mtDNA-CN:** mitochondrial DNA copy number

***MT-ND1:*** NADH-ubiquinone oxidoreductase chain 1 gene

**OS:** overall survival

**P_50_:** median

**RFS:** relapse-free survival

**ROS:** reactive oxygen species

**RTL:** relative telomere length

**RT-qPCR:** real-time quantitative polymerase chain reaction

***s*:** standard deviation

**TNM:** Tumor-Node-Metastasis system

**UICC:** Union for International Cancer Control

$\bar{\mathbf{x}}$**:** arithmetic mean

**ρ =** Spearman’s rho correlation coefficient

# References

1. Vandenbroucke JP, von Elm E, Altman DG, et al. Strengthening the reporting of observational studies in epidemiology (STROBE): Explanation and elaboration. *PLoS Med*. 2007;4(10):e297. doi:10.1371/journal.pmed.0040297

2. Gentiluomo M, Katzke VA, Kaaks R, et al. Mitochondrial DNA copy-number variation and pancreatic cancer risk in the prospective EPIC cohort. *Cancer Epidemiology, Biomarkers & Prevention*. 2020;29(3):681-686. doi:10.1158/1055-9965.EPI-19-0868

3. Cawthon RM. Telomere length measurement by a novel monochrome multiplex quantitative PCR method. *Nucleic Acids Res*. 2009;37(3):e21-e21. doi:10.1093/nar/gkn1027

4. Pfaffl MW. A new mathematical model for relative quantification in real-time RT-PCR. *Nucleic Acids Res*. 2001;29(9):45e-445. doi:10.1093/nar/29.9.e45

5. Union for International Cancer Control (UICC). *TNM Classification of Malignant Tumours*. 8th edition. (Brierley JD, Gospodarowicz MK, Wittekind C, eds.). John Wiley & Sons, Ltd; 2017.

6. World Health Organization. *International Statistical Classification of Diseases and Related Health Problems, 10th Revision (ICD-10)*. Vol 1. 5th edition. World Health Organization; 2016.

7. Umar A, Boland CR, Terdiman JP, et al. Revised Bethesda Guidelines for hereditary nonpolyposis colorectal cancer (Lynch syndrome) and microsatellite instability. *JNCI Journal of the National Cancer Institute*. 2004;96(4):261-268. doi:10.1093/jnci/djh034
